# Systematic analyses of a novel lncRNA‐associated signature as the prognostic biomarker for Hepatocellular Carcinoma

**DOI:** 10.1002/cam4.1541

**Published:** 2018-05-15

**Authors:** Jing Sui, Yan Miao, Jiali Han, Hongmei Nan, Bo Shen, Xiaomei Zhang, Yan Zhang, Yuan Wu, Wenjuan Wu, Tong Liu, Siyi Xu, Sheng Yang, Lihong Yin, Yuepu Pu, Geyu Liang

**Affiliations:** ^1^ Key Laboratory of Environmental Medicine Engineering Ministry of Education School of Public Health Southeast University Nanjing Jiangsu China; ^2^ Department of Epidemiology Richard M. Fairbanks School of Public Health, Melvin and Bren Simon Cancer Center Indiana University Indianapolis IN USA; ^3^ Department of Oncology Jiangsu Cancer Hospital Nanjing Jiangsu China

**Keywords:** Hepatocellular carcinoma, long noncoding RNA, overall survival, prognostic biomarker, The Cancer Genome Atlas

## Abstract

Accumulating evidence implies that long noncoding RNAs (lncRNAs) play a crucial role in predicting survival for Hepatocellular carcinoma (HCC) patients. This study aims to capture the current research hotspots of HCC, based on the analysis of publications related to HCC research from 2013 to 2017, and to identify a novel lncRNA signature for HCC prognosis through the data mining in The Cancer Genome Atlas (TCGA). “Prognosis” and “biomarker” were located in the core of the HCC research hotspot. Moreover, long noncoding RNA was the top one research frontier in HCC research. The associations between survival outcome and the expression of lncRNAs were evaluated by the univariate and multivariate Cox proportional hazards regression analyses. Four lncRNAs (LINC00261, TRELM3P, GBP1P1, and CDKN2B‐AS1) were identified as significantly correlated with overall survival (OS). These four lncRNAs were gathered as a single prognostic signature. There was a significant positive correlation between HCC patients with low‐risk scores and overall survival (HR = 1.802, 95%CI [1.224‐2.652], *P *= .003). Further analysis suggested that the prognostic value of this four‐lncRNA signature was independent in clinical features. The enrichment analysis of prognostic lncRNA‐related gene was performed to find out the related pathways. Our study indicates that this novel lncRNA expression signature may be a useful biomarker of the prognosis for HCC patients, based on bioinformatics analysis.

## 1 INTRODUCTION

Hepatocellular carcinoma (HCC) ranks sixth in the list of most commonly occurring solid cancers worldwide and ranks second in the list of most prevalent cause of death from fatal cancer.^1^ Hepatitis B or Hepatitis C Virus infection, alcohol drinking, and excessive smoking are the primary causes of HCC.^2^, ^3^ Despite emerging evidence in the understanding of molecular mechanisms of HCC and improved therapies for HCC, the average survival time is still short. Regarding the recent research, over 60% of initial detection of HCC patients in Japan is an early stage with an approximately 40% five‐year survival rate and an average survival time of 50 months.^4^

In the past decade, progress in the genome‐wide analysis of mammalian transcriptome has indicated a novel class of transcript, long noncoding RNAs (lncRNAs), which are broadly transcribed in the genome.^5^ LncRNAs are restricting defined as transcripts of >200 nucleotides in length, which lack significant open reading frames (ORF).^6^ In the nucleus, lncRNAs primarily modulate gene transcription and mRNA splicing, while they are involved in RNA activation and stability of miRNA in the cytoplasm.^7^

Further evidence suggests that the aberrant expressions of lncRNAs have a clinical influence on the diagnosis and prognosis of HCC.^8^, ^9^, ^10^ Till now, lncRNA‐associated biomarkers for diagnosis of HCC have been reported in many studies. Nevertheless, limited attempts have made to report the lncRNA signature as the prognostic biomarkers for HCC patients.

This study aims to capture the current research hotspots of HCC, based on the analysis of publications related to HCC research from 2013 to 2017, and to identify a novel lncRNA signature for HCC prognosis through the data mining in The Cancer Genome Atlas (TCGA) (http://cancergenome.nih.gov). Through constructing a comprehensive lncRNA expression analyses, we identified a new candidate indicator for the overall survival (OS) prediction in HCC patients.

## 2 METHODS AND MATERIALS

### 2.1 Source of the literature data and search strategy

Literature was searched from the Science Citation Index‐Expanded (SCI‐E) of Web of Science (WOS) of Clarivate Analytics on June 30, 2017. The data were collected from the public database, did not involve any interactions with human or animal subjects. Ethical approval was not applicable here.

All searches were conducted on the same day, June 30, 2017, to avoid the bias of daily updating of the database. The following terms were used in search: Title =  (“liver cancer*”) OR Title = (“liver neoplasm*”) OR Title =  (“Hepatocellular Cancer*”) OR Title =  (“Hepatocellular carcinoma*”) OR Title =  (“hepatic cancer*”) OR Title =  (“hepatic neoplasm*”) OR Title =  (“cancer of the liver”) OR Title =  (“cancer of liver”) AND Language = English. In this case, only research articles and review articles were included.

### 2.2 Literature data collection and analysis method

The data were independently collected from all eligible publications by two authors (Jing Sui and Yan Miao). The txt data were downloaded from WOS, and were imported into VOSviewer 1.6.5 (Leiden University, Leiden, Netherlands) and CiteSpace V (Drexel University, Philadelphia, PA, USA). The data were analyzed objectively. VOSviewer was performed to carry out the cluster analysis of the literature and the hotspot analysis of keywords.

### 2.3 TCGA database and patient information

Three hundred and seventy‐seven HCC patients’ data were downloaded from TCGA database (up to January 28, 2016). After exclusion criteria: (1) histologic diagnosis ruled out HCC; (2) another malignancy besides HCC. Overall, 317 HCC patients with corresponding clinical features such as race, age, gender, tumor stage, radiation therapy, and residual tumor were included in this study. Moreover, the endpoint in this study was OS. Of these above 317 HCC patients, there were 154 HCC patients with tumor stage I, 78 HCC patients with tumor stage II, 80 HCC patients with tumor stage III, and 5 HCC patients with tumor stage IV. As the data were retrieved from the public database (TCGA database), further ethical approval was not applicable in this study. Data processing procedures also met the policy of TCGA data and human subject protection (http://cancergenome.nih.gov/publications/publicationguidelines).

### 2.4 RNA sequence data procession and lncRNA profile mining

**Figure 1:**
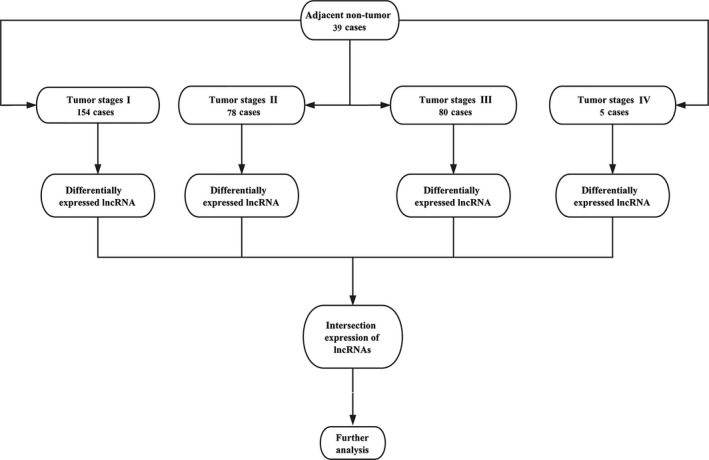
Flowchart of bioinformatics analysis

The HCC RNA level 3 expression data were downloaded from TCGA database. All the lncRNA sequencing raw reads were postprocessed and normalized using TCGA RNASeqv2 system.^11^ In this study, lncRNAs with a description from NCBI (https://www.ncbi.nlm.nih.gov/gene/) and Ensemble (http://www.ensembl.org/index.html) would be selected for further study. To identify the differential expression of lncRNAs, patients were divided into HCC four tumor stages, including I, II, III, and IV to compare with adjacent nontumor lung tissues, respectively. The intersection of lncRNAs was selected in the further analysis (Figure 1).

### 2.5 Construction of the lncRNA‐based prognostic signature and Statistical analysis

The expression profile of each lncRNA was normalized by log2‐transformed for further statistical analysis. However, the differently expressed lncRNAs that were 0 in more than 10% of all data were eliminated. The univariate Cox proportional hazards regression was used to evaluate the association between the differently expressed lncRNAs with OS of HCC patients (*P*‐value <.05). Then, the multivariate Cox proportional hazards regression was used to identify the prognostic value of these independent lncRNA biomarkers. Meanwhile, the prognostic lncRNA signature (the risk score model) was constructed based on a combination of the expression profiles of each prognostic lncRNAs, weighted by their estimated regression coefficients in the multivariate Cox proportional hazards regression analysis as follows: risk score = exp_lncRNA1_*β_lncRNA1_ + exp_lncRNA2_*β_lncRNA2_ + … exp_lncRNAn_*β_lncRNAn_.

The Kaplan‐Meier survival curves were performed to present the difference in OS between high‐risk score group and low‐risk score group. The statistical significance was examined by the log‐rank test. The univariate and multivariate Cox proportional hazards regressions for OS were conducted for individual clinical features with the lncRNA signature. The hazard ratio (HR) and 95% confidence intervals (CI) were calculated in this study. The prognostic performance at five years was accessed using time‐dependent receiver operating characteristic (ROC) curves.^12^

### 2.6 Functional enrichment analysis

To investigate the biological feature of these above four lncRNAs in lncRNA signature, we identified the genes that highly correlated with these above four lncRNAs expression (Pearson |R| > 0.5) in TCGA database. Pathways and biological processes were predicted using functional enrichment analysis of Gene Ontology (GO) and the Kyoto Encyclopedia of Genes and Genomes (KEGG) in the Database for Annotation, Visualization, and Integrated Discovery (DAVID) (https://david.ncifcrf.gov/) Bioinformatics Resources 6.8.^13^, ^14^ The *P*‐value <.05 and FDR <0.05 were considered to be significant. Subsequently, the protein‐protein interaction (PPI) network was constructed with the coexpressed genes via STRING (https://string-db.org/).^15^, ^16^

## 3 RESULTS

### 3.1 Cluster analysis and hotspot analysis on HCC research

**Figure 2:**
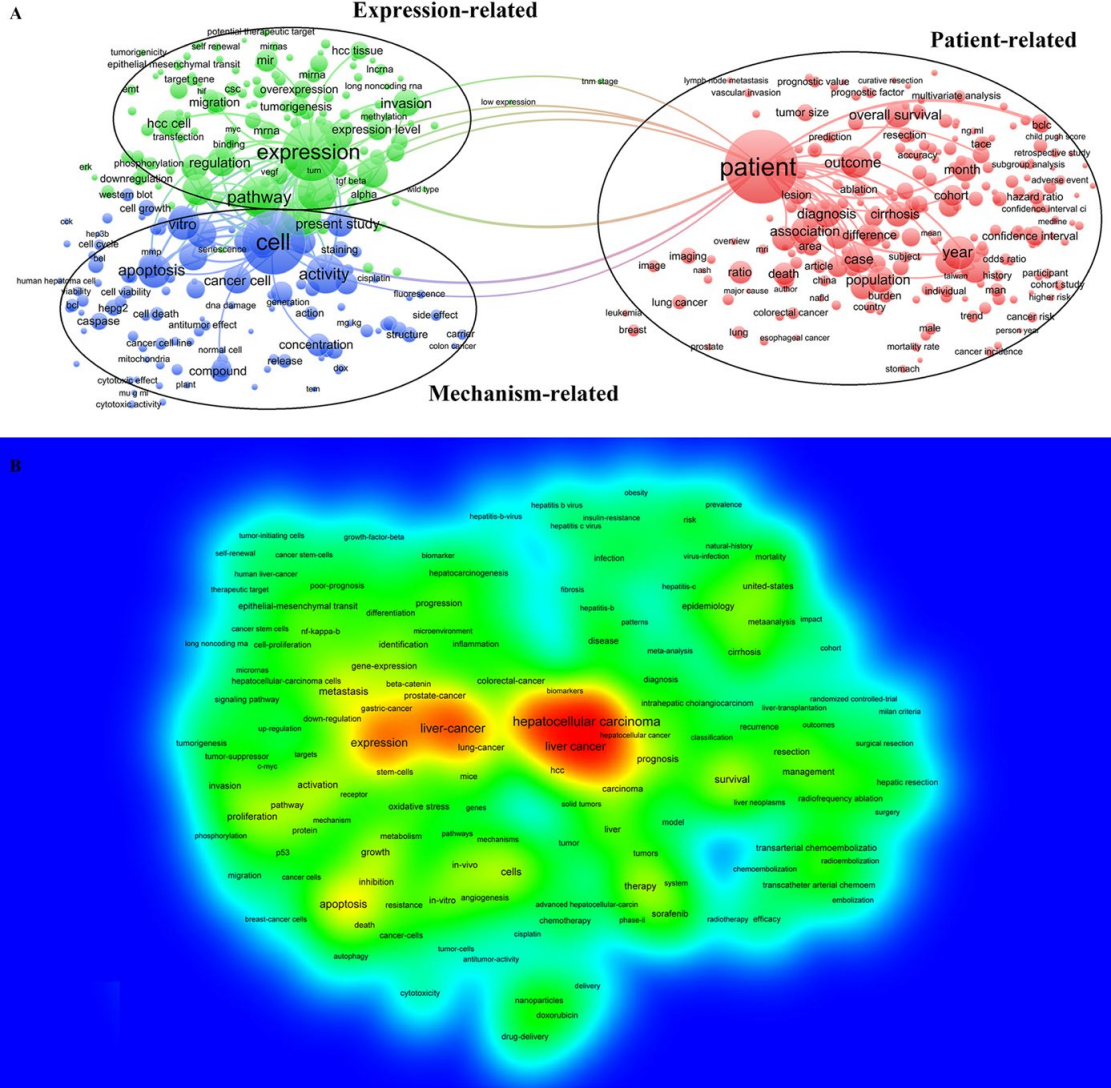
Cluster analysis and hotspot analysis on Hepatocellular carcinoma research. A, The divided into three clusters: “Patients Related Study,” “Expression Related Study,” and “Cell Related Study.” The cluster analysis demonstrated that the dominant fields of Hepatocellular carcinoma include three research directions. B, Keywords with high frequency were captured and considered as the hotspots in this field

A total of 1792 papers met the search criteria. These papers were analyzed by VOSviewer and divided into three clusters: “Patients Related Study,” “Expression Related Study,” and “Cell Related Study.” The cluster analysis demonstrated that the dominant fields of HCC include three research directions (Figure 2A).

Keywords used in the 1792 papers were extracted and analyzed by VOSviewer. As shown in Figure 2B, VOSviewer applied colors to keywords. The color of an item was determined by the frequency of occurrence, where by default colors range from blue (low frequency) to green (median frequency) to red (high frequency). Keywords with high frequency were captured and considered as the hotspots in this field. From the literature analysis, we found hot keywords, including Hepatocellular carcinoma, prognosis, and biomarker. Thus, we confirmed that the current research hotspot of HCC is to identify a prognostic‐biomarker for HCC.

**Figure 3:**
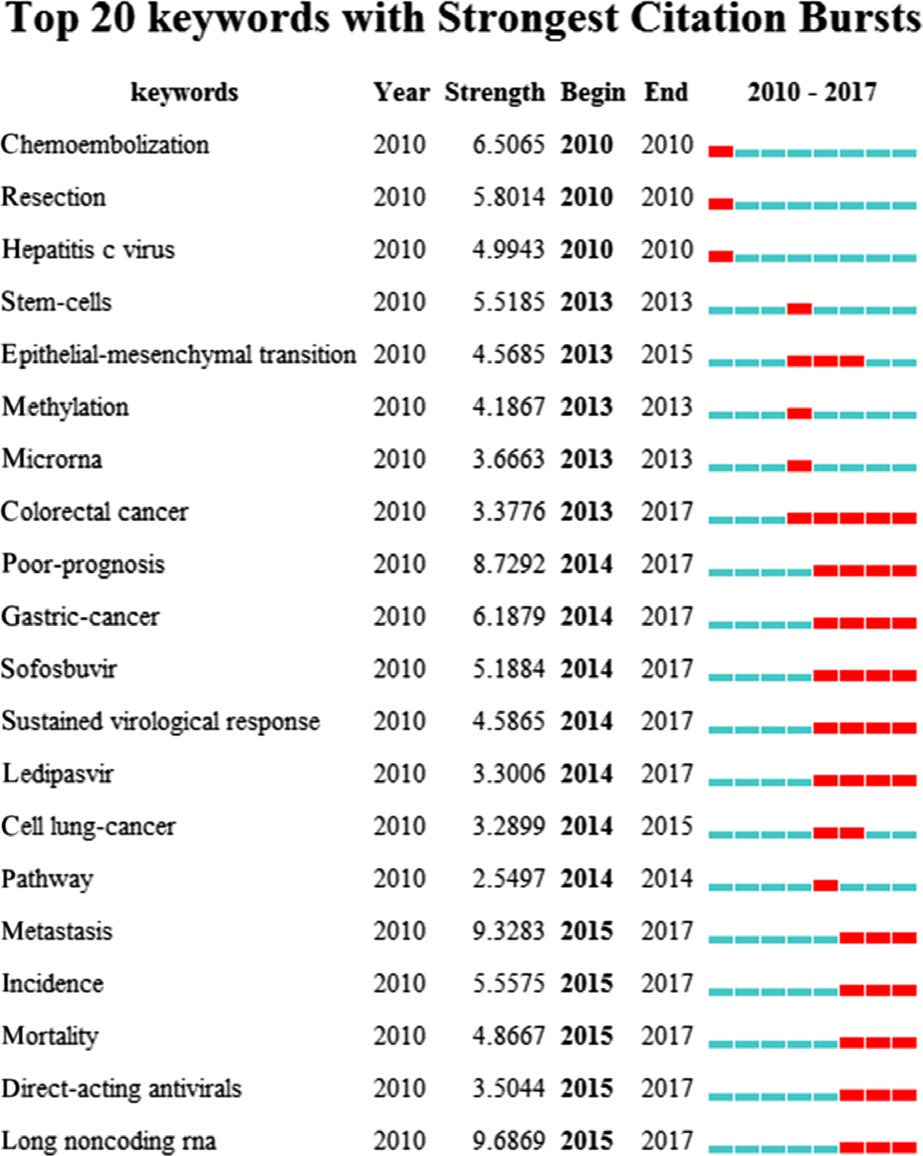
The keywords with the strongest citation bursts of publications on Hepatocellular carcinoma research

Furthermore, CiteSpace V was performed to capture the keywords with the most energetic citation bursts that identified as research frontiers over time. The top one research frontier of HCC research was “long noncoding RNA” (Figure 3). We realized a keyword “long noncoding RNA” appeared and grew rapidly. Considering this, our team determined the final research objective that was to discover a lncRNA‐related prognostic biomarker for HCC. Based on this destination, we proceeded to the next step of lncRNA‐related data mining. Here, we chose The Cancer Genome Atlas (TCGA) as a data source for both clinical information and bio‐information.

### 3.2 Patient characteristics

There were 317 HCC patients included in this study downloaded from TCGA dataset. Based on American Joint Committee on Cancer (AJCC) TNM stage, the HCC patients were divided into stage I, stage II, stage III and stage IV, four groups. The age of all HCC patients was 58.019 ± 13.509 years. The OS time was 813.108 ± 747.979 days, 106 of 317 (33.438%) HCC patients died.

### 3.3 Identification of differentially expressed lncRNAs

**Figure 4:**
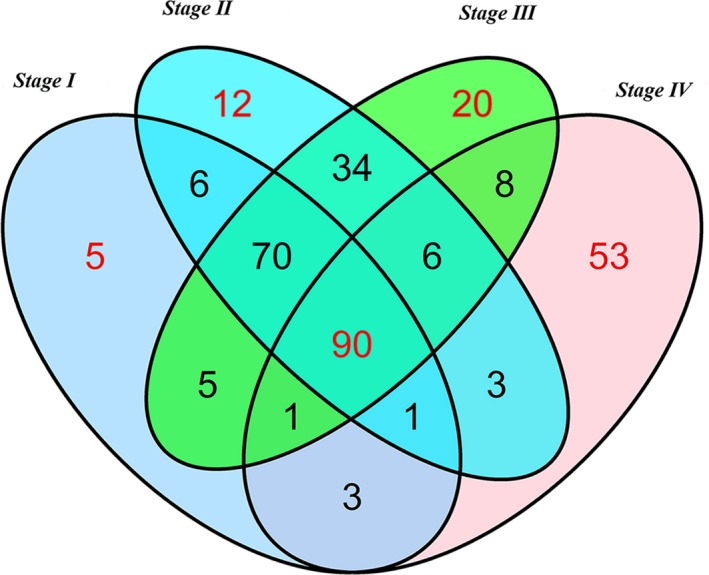
Venn diagram analysis of differentially expressed lncRNA in Hepatocellular carcinoma. Each ellipse represents a tumor stage group. The RNA in the middle represents significantly and consistently differentially expressed in four groups

**Figure 5:**
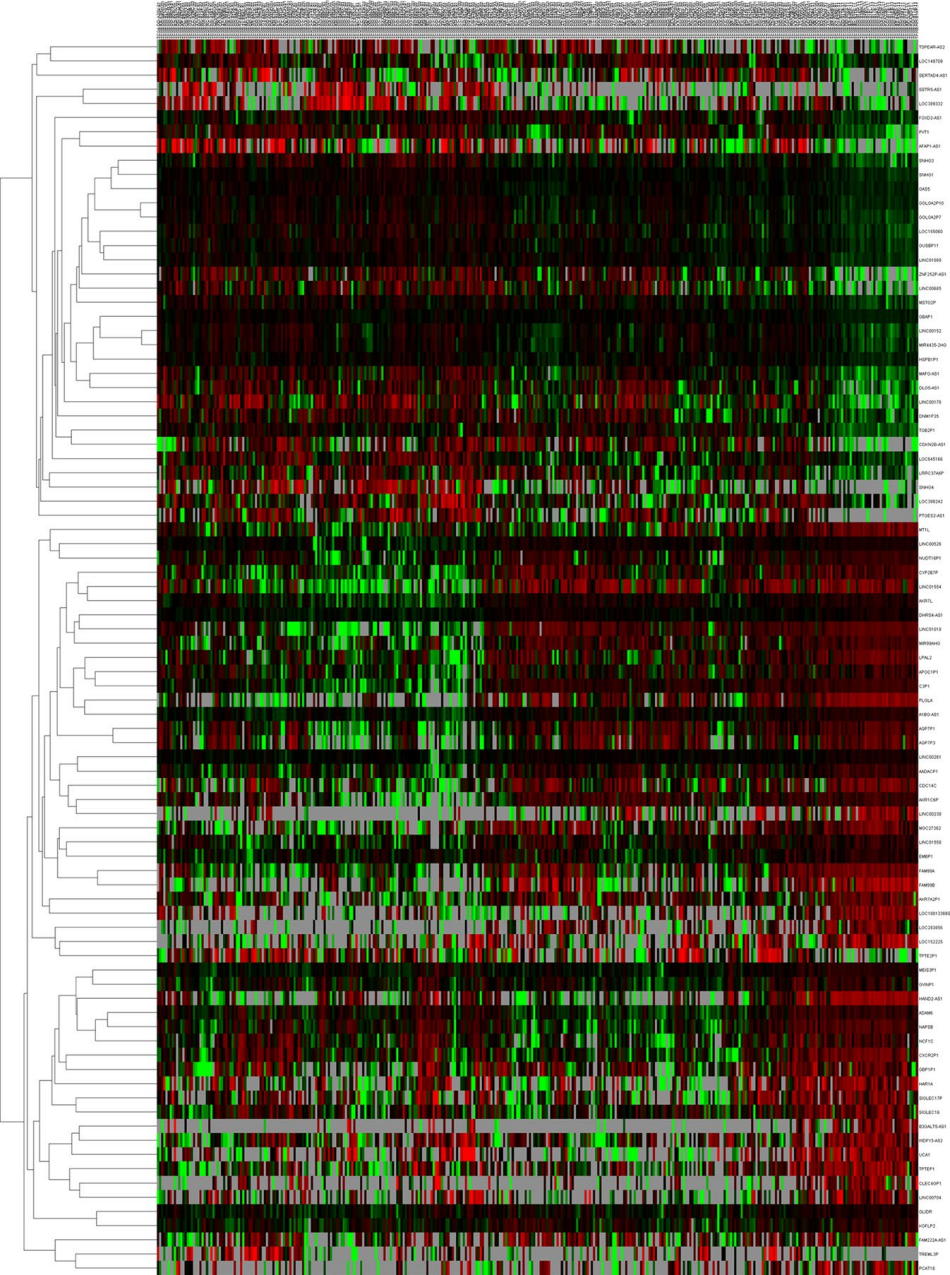
The differential expression of intersected lncRNAs in Hepatocellular carcinoma. A heatmap is showing the differentially expressed lncRNAs

We performed differential expression analysis by comparing the expression of 1081 lncRNAs in HCC and adjacent nontumor liver tissues. Fold change>2 or <0.5, *P*‐value <.05 and FDR <0.05 were set up to identify significantly differentially expressed lncRNAs. Three hundred and seventeen differentially expressed lncRNAs were selected for further analysis, including 181 lncRNAs in stage I, 222 lncRNAs in stage II, 234 lncRNAs in stage III, and 165 lncRNAs in stage IV. We combined these four groups of 317 differentially expressed lncRNAs together, and 90 lncRNAs were identified stability differentially expressed in all of the HCC tumor stages via two methods (Figures 4 and 5). The differentially expressed lncRNAs in different tumor stages were shown in Table S1.

### 3.4 Prognostic signature construction

**Figure 6:**
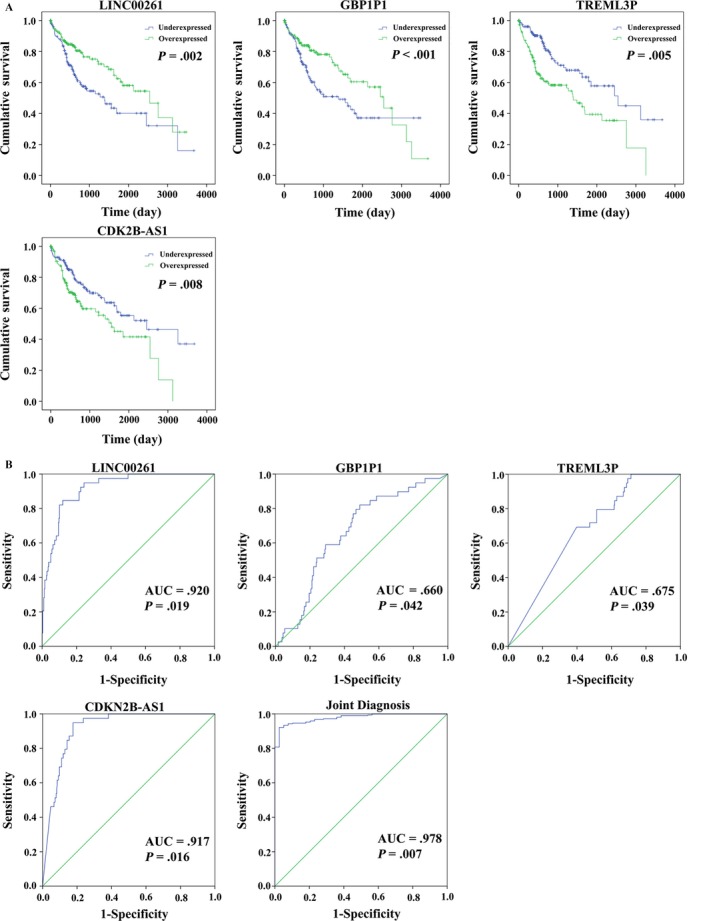
Four differentially expressed lncRNAs (LINC00261, TRELM3P, GBP1P1, and CDKN2B‐AS1). A, Kaplan‐Meier curves showing the relationship between these four lncRNAs and overall survival. The patients were divided into over‐ and underexpression groups by the mean lncRNAs level; B, ROC curves of the four lncRNAs to distinguish HCC tissue from adjacent normal tissues

Based on these 165 differentially expressed lncRNAs and clinical features in 317 HCC patients from TCGA database, 18 lncRNAs significantly associated with OS (*P *< .05) were identified by the univariate Cox regression model in Table 1. Afterward, the multivariate Cox proportional hazards regression analysis was used to calculate the interrelated relationship among 18 lncRNAs with OS, and only four lncRNAs exhibited a significant prognostic value for HCC, including LINC00261, TRELM3P, GBP1P1 and CDKN2B‐AS1 (Table 2 and Figure 6).

**Table 1:** Prognostic value of the differentially expressed lncRNAs by univariate cox regression analysis

LncRNA	Estimate	StdErr	ChiSq	*P*	Hazard ratio (95%CI)
AADACP1	−0.539	0.199	7.335	**.007** a	0.58 (0.395‐0.862)
C3P1	−0.451	0.197	5.237	**.022** a	0.637 (0.433‐0.937)
CDKN2B‐AS1	0.522	0.198	6.957	**.008** a	1.686 (1.144‐2.486)
DHRS4‐AS1	−0.536	0.198	7.343	**.007** a	0.585 (0.397‐0.862)
FOXD2‐AS1	0.459	0.197	5.449	**.020** a	1.583 (1.076‐2.329)
GBP1P1	−0.546	0.199	7.558	**.006** a	0.579 (0.392‐0.855)
GOLGA2P7	0.444	0.196	5.123	**.024** a	1.559 (1.061‐2.290)
GVINP1	−0.394	0.197	4.014	**.045** a	0.675 (0.459‐0.992)
LINC00152	0.636	0.200	10.119	**.001** a	1.889 (1.277‐2.796)
LINC00261	−0.604	0.200	9.144	**.002** a	0.547 (0.370‐0.809)
LINC01018	−0.398	0.197	4.089	**.043** a	0.672 (0.457‐0.988)
LINC01554	−0.450	0.199	5.110	**.024** a	0.638 (0.432‐0.942)
LOC645166	0.507	0.198	6.563	**.010** a	1.660 (1.126‐2.445)
MAFG‐AS1	0.423	0.196	4.645	**.031** a	1.526 (1.039‐2.241)
MEIS3P1	−0.474	0.197	5.820	**.016** a	0.622 (0.423‐0.915)
PLGLA	−0.497	0.200	6.202	**.013** a	0.608 (0.411‐0.899)
TREML3P	0.795	0.203	15.314	**<.001** a	2.214 (1.487‐3.296)
TSPEAR‐AS2	−0.498	0.197	6.394	**.011** a	0.608 (0.413‐0.894)

Bold font represents a statistically significant p‐value.

a *P *< .05.

**Table 2:** Prognostic value of the differentially expressed lncRNAs by multivariate Cox regression analysis

LncRNA	Estimate	StdErr	ChiSq	*P*	HR (95%CI)
LINC00261	−0.511	0.203	6.332	**.012** a	0.600 (0.403‐0.893)
TREML3P	0.671	0.206	10.638	**.001** a	1.957 (1.307‐2.930)
GBP1P1	−0.554	0.200	7.671	**.006** a	0.575 (0.388‐0.850)
CDKN2B‐AS1	0.447	0.200	5.005	**.025** a	1.564 (1.057‐2.314)

HR, hazard ratio; CI, confidence interval.

Bold font represents a statistically significant p‐value.

a *P *< .05.

The risk score for predicting prognostic value was constructed with the formula:

$$\begin{array}{cc} & \text{Risk score}\;=\;\\ & {\text{Exp}}_{\mathrm{LINC}00261}*\left(-0.511\right)\;+\;{\text{Exp}}_{\mathrm{TREML}3P}*\left(0.671\right)\\ & \;+\;{\text{Exp}}_{\mathrm{GBP}1P1}*\left(-0.554\right)+{\text{Exp}}_{\mathrm{CDKN}2B-\mathrm{AS}1}*\left(0.447\right). \end{array}$$

**Figure 7:**
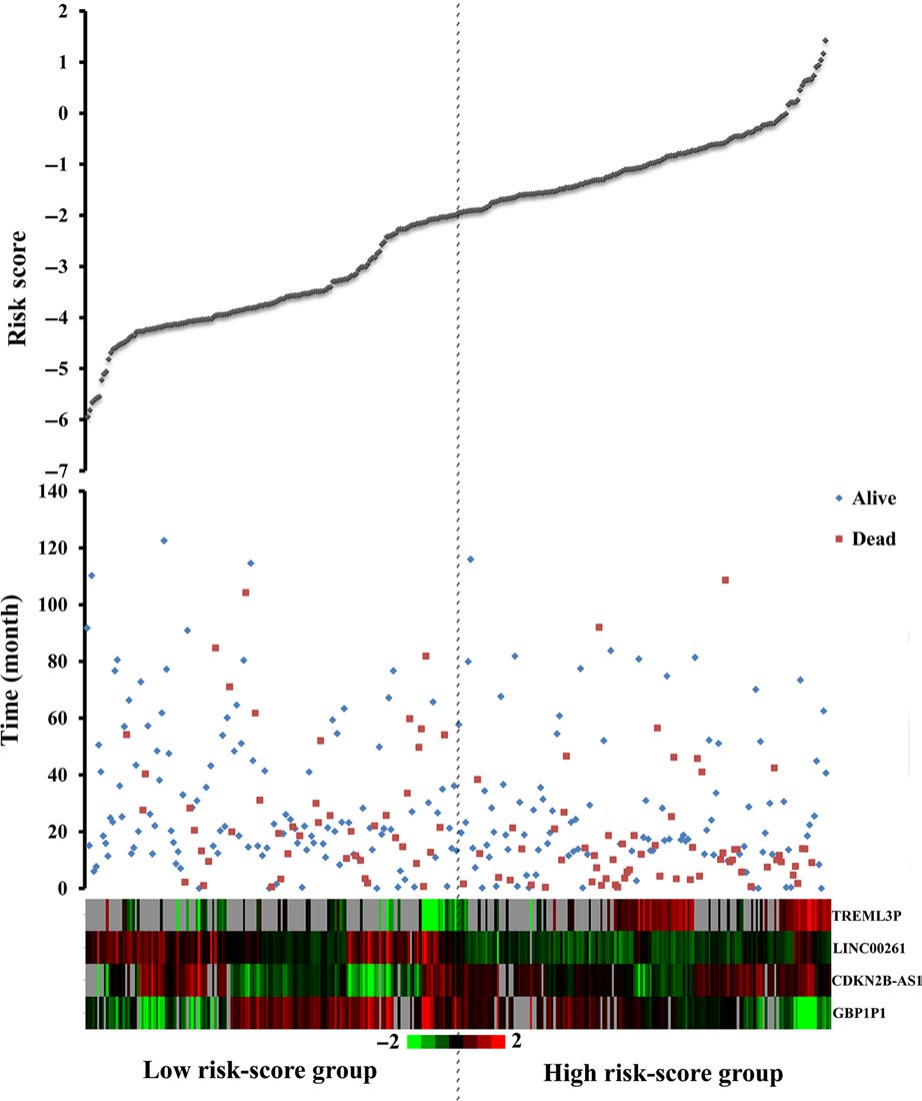
Risk score analysis of the differentially expressed lncRNA signature of Hepatocellular carcinoma. Survival status and duration of cases (Top); risk score of lncRNA signature (Middle); low and high score groups for the four lncRNAs (Bottom). Color from green to red means the expression level of lncRNAs from low to high. The dotted line indicates the median inflection point of the risk score curve, by which the Hepatocellular carcinoma patients were divided into the low‐risk and high‐risk group

**Figure 8:**
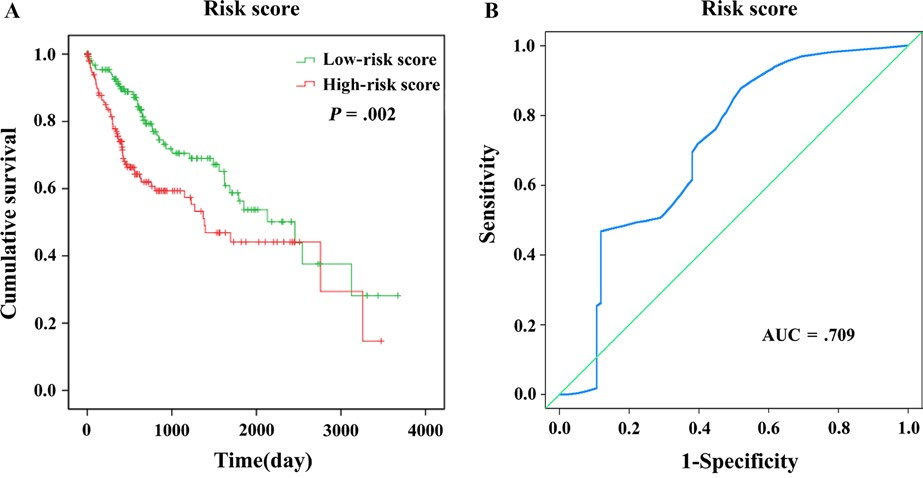
The four differentially expressed lncRNA signature of Hepatocellular carcinoma for the outcome. A, The Kaplan‐Meier test of the risk score for the OS. B, The risk score is shown by the time‐dependent ROC curve for predicting 5‐year survival

Based on the risk score model, HCC patients were classified as low‐risk score or high‐risk score patients via the median risk score as the cutoff value, which divided into the low‐risk score group (n = 159) and high‐risk score group (n = 158) (Figure 7). K‐M curves confirmed that the survival time of patients in the low‐risk score group was 929.698 ± 773.779 days, predominantly longer than that of the high‐risk score group (695.032 ± 703.854 days, *P *= .002, Figure 8A). Furthermore, the risk score could largely predict the 5‐year survival of HCC patients, as the area under ROC curve (AUC) was 0.709 (Figure 8B).

**Figure 9:**
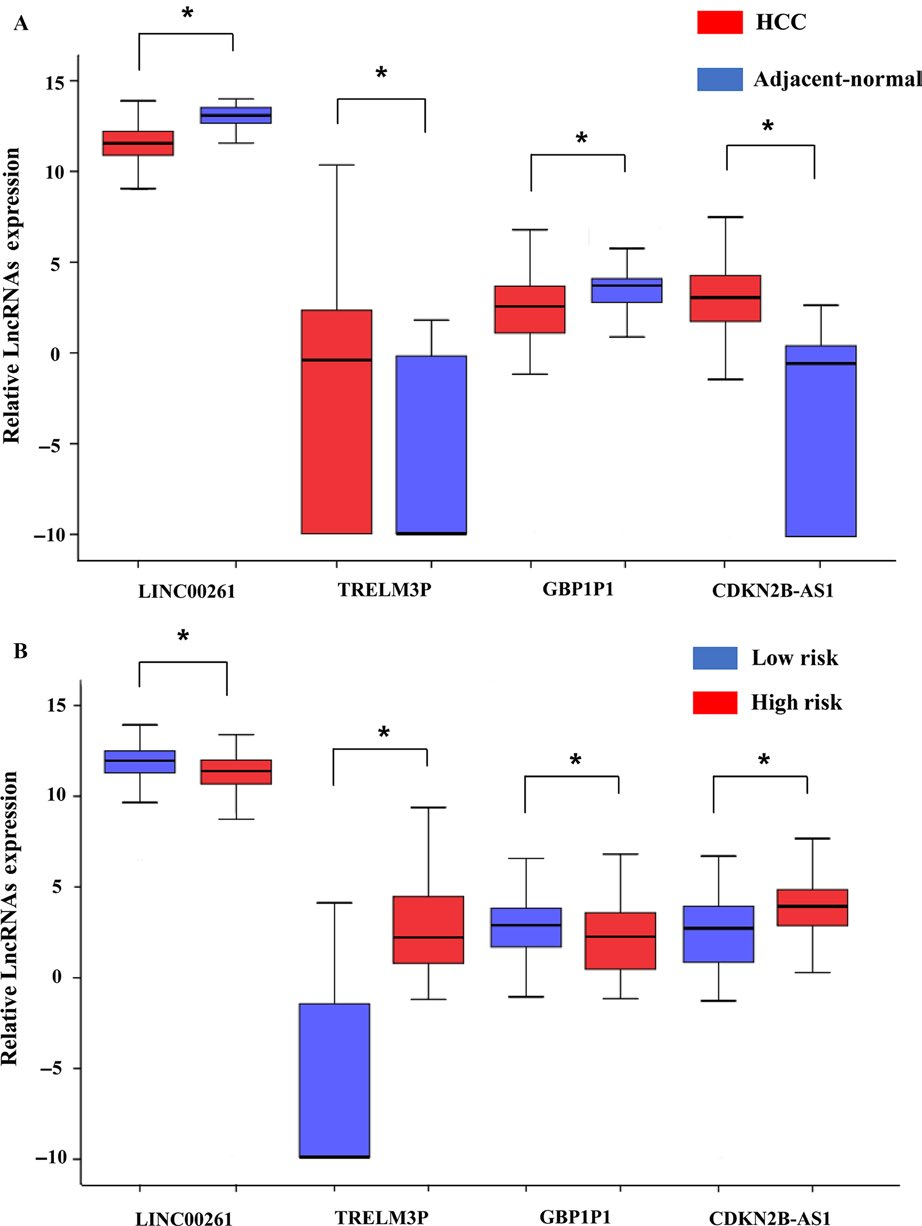
The expression level of the four lncRNAs (LINC00261, TRELM3P, GBP1P1, and CDKN2B‐AS1). A, The expression level of lncRNAs between Hepatocellular carcinoma tissues and adjacent normal tissues. B, The expression level of lncRNAs between the low‐risk and high‐risk groups. *P < .05

The expression pattern of these four differentially expressed lncRNAs in the HCC and adjacent normal tissues, low‐risk score and high‐risk score groups were shown in Figure 9.

### 3.5 Correlation between lncRNA signature and clinical characteristics

We examined the association of four‐lncRNA signature (risk score) with clinical features in HCC patients used the univariate and multivariate Cox proportional hazard regression analysis. The univariate Cox proportional hazards regression showed that gender, TNM stage, T stage, M stage, Neoplasm cancer (person neoplasm cancer status), BMI and history of Hepatocellular Carcinoma risk factors (Hist hepato carc fact) could predict poorer survival of HCC patients in Table 3 (*P *< .05). Meanwhile, the multivariate Cox proportional hazards regression showed Neoplasm cancer (*P *= .002) and risk score (*P *< .001) could predict as an independent prognostic indicator of HCC (Table 3).

**Table 3:** The predictive values of related clinical features and risk score

Variables	PatientN = 317	Univariate analysis		Multivariate analysis	
		HR (95% CI)	*P*	HR (95% CI)	*P*
Race					
Asian	151	1 (reference)			
Black	14	1.890 (0.746‐4.793)	.180		
White	141	1.138 (0.757‐1.710)	.535		
Gender					
Female	99	1 (reference)		1 (reference)	
Male	217	0.657 (0.445‐0.969)	**.034** a	1.354 (0.698‐2.626)	.370
Age					
<=55	119	1 (reference)			
>55	197	1.102 (0.739‐1.644)	.634		
TNM stage					
I	154	1 (reference)		1 (reference)	
II	77	1.339 (0.799‐2.244)	.268	1.636 (0.802‐3.338)	.176
III	80	2.592 (1.668‐4.028)	<**.001** a	2.714 (1.467‐5.019)	**.001** a
IV	5	5.499 (1.689‐17.901)	**.005** a		
T stage					
T1	156	1 (reference)		1 (reference)	
T2	79	1.294 (0.774‐2.163)	.325	0.000 (0.000‐1.640E^58^)	.908
T3	71	2.461 (1.565‐3.869)	**<.001** a	0.448 (0.051‐3.955)	.470
T4	10	5.040 (2.231‐11.384)	**<.001** a	0.617 (0.061‐6.210)	.682
N stage					
N0	243	1 (reference)			
N1	1	0.049 (0.000‐4.654E^32^)	.940		
M stage					
M0	248	1 (reference)			
M1	4	3.960 (1.243‐12.617)	**.020** a		
Radiation therapy					
No	288	1 (reference)			
Yes	8	1.074 (0.340‐3.397)	.903		
Neoplasm cancer					
Tumor free	174	1 (reference)		1 (reference)	
With tumor	126	2.498 (1.643‐3.798)	**<.001** a	2.432 (1.386‐4.267)	**.002** a
Residual tumor					
R0	280	1 (reference)			
R1 + R2	11	1.038 (0.328‐3.284)	.949		
Fibrosis ishak score					
No fibrosis	62	1 (reference)			
Portal fibrosis	28	0.861 (0.365‐2.035)	.734		
Fibrous speta	24	0.896 (0.362‐2.219)	.813		
Nodular formation and incomplete cirrhosis	8	0.841 (0.196‐3.603)	.816		
Established cirrhosis	57	0.807 (0.420‐1.552)	.521		
BMI					
<18.5	18	0.485 (0.188‐1.250)	.134	0.439 (0.138‐1.396)	.163
18.5‐23.9	128	1 (reference)		1 (reference)	
24‐27.9	70	0.505 (0.297‐0.856)	**.011** a	0.543 (0.279‐1.056)	.072
≥28	74	0.611 (0.369‐1.012)	.056	0.346 (0.159‐0.754)	**.008** a
Histologic grade					
G1	41	1 (reference)			
G2	150	1.144 (0.608‐2.155)	.677		
G3	112	1.293 (0.678‐2.469)	.436		
G4	12	1.770 (0.620‐5.053)	.286		
Platelet result					
<100 × 10^^9^	15	2.061 (0.924‐4.599)	.077		
100‐300 × 10^^9^	200	1 (reference)			
>300 × 10^^9^	44	1.674 (0.990‐2.829)	.054		
Family cancer history					
No	185	1 (reference)			
Yes	92	1.150 (0.767‐1.725)	.500		
Vascular tumor cell type					
None	178	1 (reference)			
Micro	76	1.019 (0.602‐1.725)	.944		
Macro	14	2.067 (0.933‐4.582)	.074		
Hist hepato carc fact					
No history of primary risk factors	86	1 (reference)		1 (reference)	
Alcohol consumption	95	0.649 (0.399‐1.056)	.082	0.605 (0.299‐1.223)	.162
Hepatitis b	76	0.373 (0.208‐0.671)	**.001** a	0.461 (0.214‐0.996)	**.049** a
Hepatitis c	29	0.876 (0.435‐1.764)	.712	0.389 (0.127‐2.626)	.098
Risk score					
Low	159	1 (reference)		1 (reference)	
High	157	1.802 (1.224‐2.652)	**.003** a	2.997 (1.634‐5.497)	**<.001** a

HR, hazard ratio; CI, confidence interval; BMI, Body Mass Index; Hist hepato carc fact, history of Hepatocellular Carcinoma risk factors.

Bold font represents a statistically significant p‐value.

a *P *< .05.

**Figure 10:**
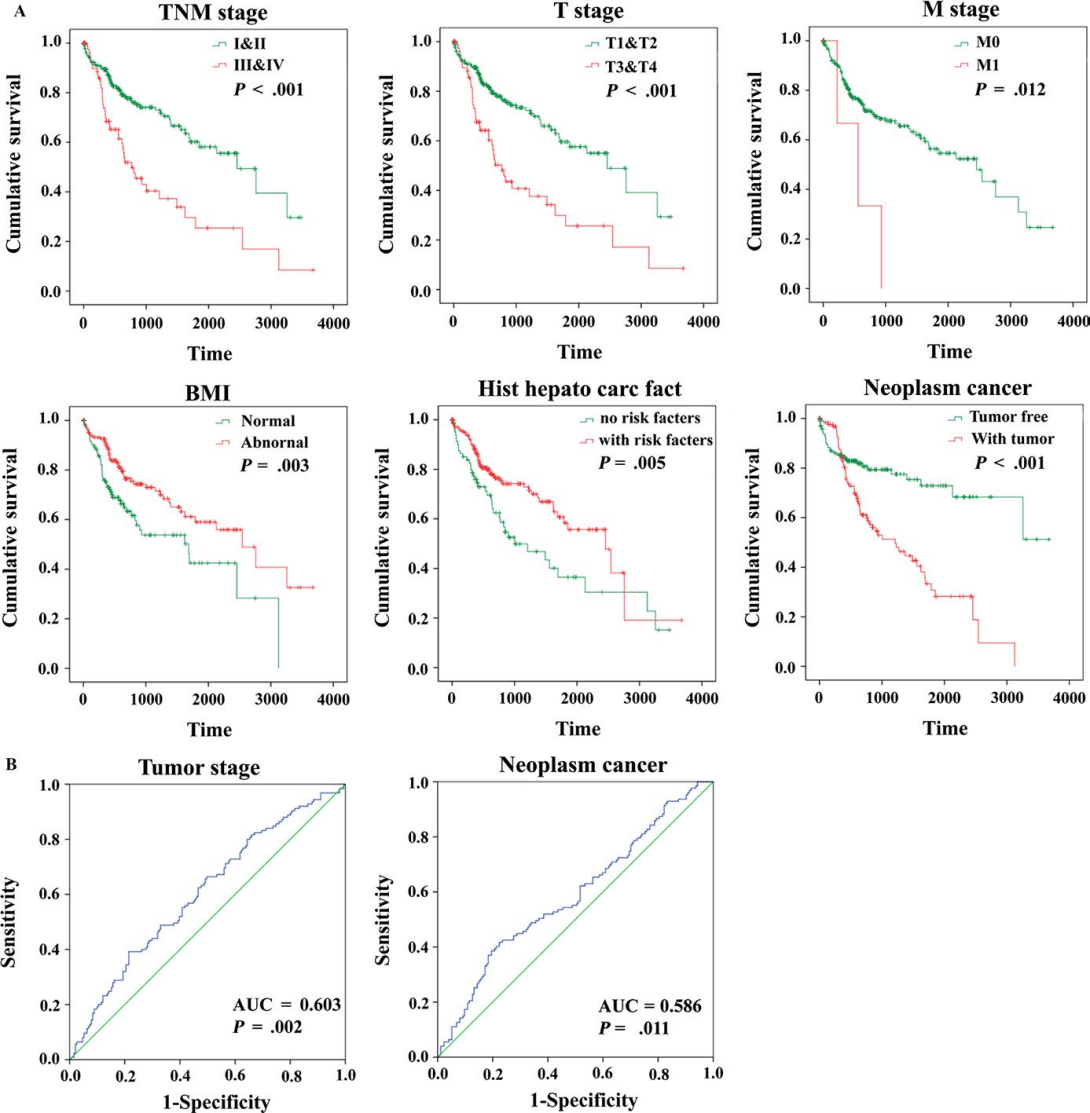
The prognostic value of different clinical features for OS and the predictive value of the risk score for clinical features of Hepatocellular carcinoma patients. A, Kaplan‐Meier curves of seven independent prognostic indictors. B, ROC curve is predicting different clinical features

In this study, the K‐M curves of these clinical features were shown in Figure 10A. Moreover, it synthetically presented that the risk score conferred a prognostic value for predicting patients’ status of tumor stage (AUC = 0.603, *P *= .002) and Neoplasm cancer (AUC = 0.586, *P *= .001) (Figure 10B).

### 3.6 Functional assessment of the four‐lncRNA signature

**Figure 11:**
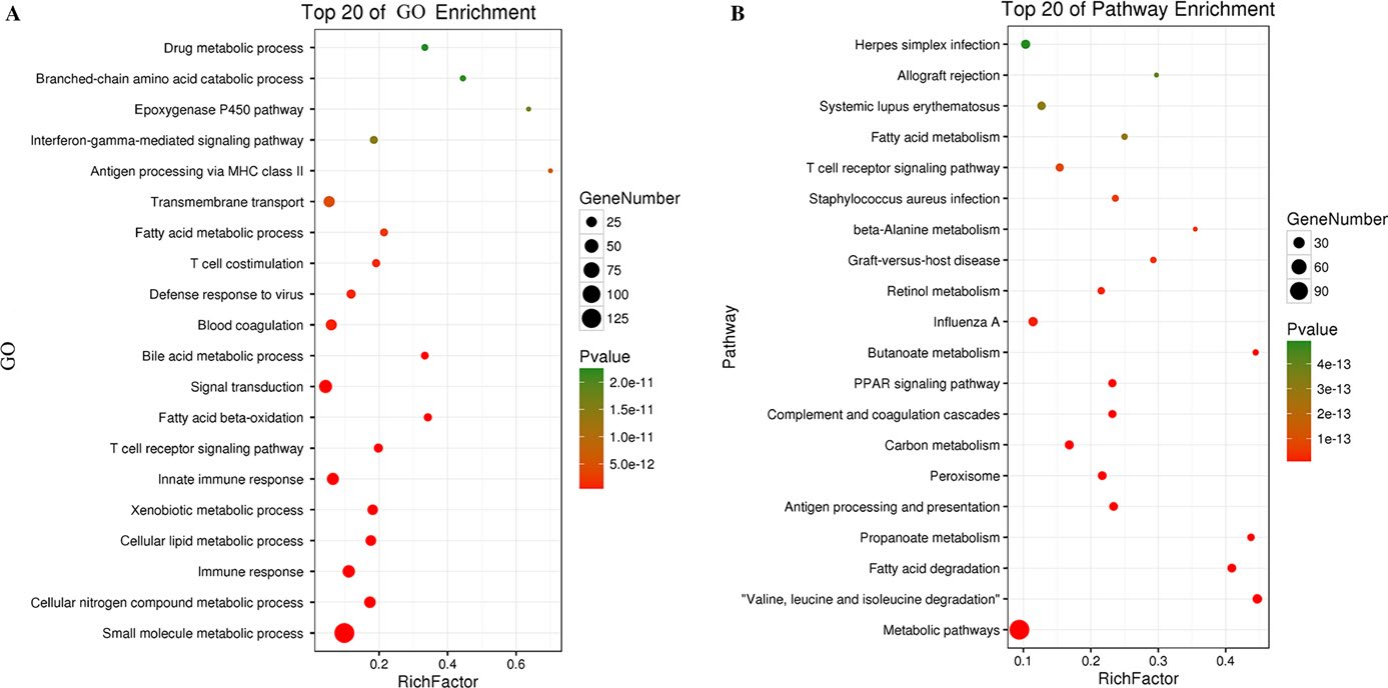
Top 20 enrichment of GO terms and KEGG pathways for coexpressed mRNAs

**Figure 12:**
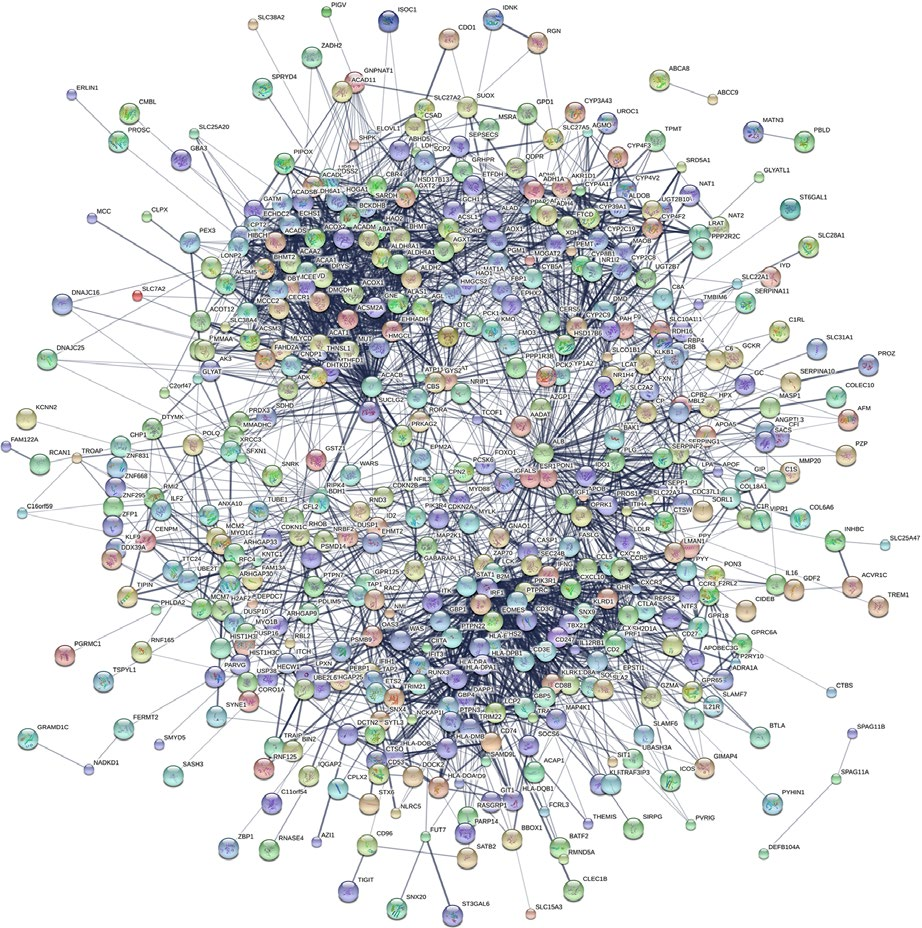
The map represents the protein‐protein interaction (PPI) network of coexpressed genes

There were 626 genes identified in TCGA database coexpressed with these four lncRNAs (LINC00261, TRELM3P, GBP1P1, and CDKN2B‐AS1) (|R|>0.5), including 424 genes with LINC00261, 36 genes with TRELM3P, 132 genes with GBP1P1, and 31 genes with CDKN2B‐AS1, respectively (Table S2). It revealed enrichment of 628 GO Terms and 131 Pathways (*P*‐value <.05 and an enrichment score of >1.5; Table S3). It was found that the top GO biological process of coexpressed genes was small molecule metabolic process (GO: 0044281) and cellular nitrogen compound metabolic process (GO: 0034641) (Table 4 and Figure 11A). After the pathway analysis, the coexpressed genes were mainly enriched in Metabolic pathways and “Valine, leucine and isoleucine degradation” (Table 4 and Figure 11B). For the construction of the protein‐protein interaction (PPI) network, there were 470 genes in the PPI network, which were regarded as hub genes (Figure 12).

**Table 4:** Top 15 KEEG pathways and GO terms enriched by the coding genes

Category	Term	No. of genes	–lgP
Go term	Small molecule metabolic process	134	87.035
	Cellular nitrogen compound metabolic process	32	27.871
	Immune response	39	26.456
	Cellular lipid metabolic process	26	22.824
	Xenobiotic metabolic process	25	22.295
	Innate immune response	36	16.620
	T‐cell receptor signaling pathway	17	15.850
	Fatty acid beta‐oxidation	13	15.568
	Signal transduction	45	14.248
	Bile acid metabolic process	12	14.211
	Blood coagulation	28	12.113
	Defense response to virus	17	11.978
	T‐cell costimulation	13	11.930
	Fatty acid metabolic process	12	11.644
	Transmembrane transport	29	11.348
	Antigen processing via MHC class II	7	11.208
	Interferon‐gamma‐mediated signaling pathway	12	10.823
	Epoxygenase P450 pathway	7	10.773
	Branched‐chain amino acid catabolic process	8	10.660
	Drug metabolic process	9	10.649
KEGG pathways	Metabolic pathways	116	72.555
	“Valine, leucine and isoleucine degradation”	21	28.145
	Fatty acid degradation	18	23.249
	Propanoate metabolism	14	18.589
	Antigen processing and presentation	18	18.187
	Peroxisome	18	17.552
	Carbon metabolism	19	16.314
	PPAR signaling pathway	16	16.109
	Complement and coagulation cascades	16	16.109
	Butanoate metabolism	12	16.031
	Influenza A	20	13.807
	Retinol metabolism	14	13.615
	Graft‐versus‐host disease	12	13.432
	Beta‐Alanine metabolism	11	13.376
	Staphylococcus aureus infection	13	13.211
	T‐cell receptor signaling pathway	16	13.113
	Fatty acid metabolism	12	12.515
	Systemic lupus erythematosus	17	12.501
	Allograft rejection	11	12.396
	Herpes simplex infection	19	12.311

## 4 DISCUSSION

Hepatocellular carcinoma (HCC) is one of the deadliest malignancies with the high global mortality. Most HCC patients were diagnosed in the advanced stages of tumor progression (stage III and stage IV).^17^ However, HCC patients in the same stage might exhibit different prognosis outcome, owning to differences in various biomarkers, which are still being discovered.^18^ The novel biomarkers for early diagnosis, therapeutic process monitoring, and prognostic evaluation might increase the survival rate for HCC. Accumulating evidence suggested that lncRNAs might play major role in tumorigenesis, metastasis, development and the prognosis of HCC.^19^, ^20^, ^21^, ^22^ The large‐scale genome analyses have revealed the molecular characteristics associated with HCC OS.^23^, ^24^, ^25^ However, most studies focused on miRNA, mRNA, gene, and protein expression.^26^, ^27^, ^28^, ^29^, ^30^ With knowledge growing, the functional role of lncRNAs in tumorigenesis and development also represents a significant untapped resource for HCC prognosis.

In the present study, to identify lncRNAs significantly related to the OS of HCC, HCC data were analyzed on HCC patients TNM stage with clinical features from the TCGA database in groups. After the univariate and multivariate Cox proportional hazards regression, a total of four HCC OS‐related lncRNAs were identified as significant prognostic value for HCC survival. Then, the signature (risk score) was set by combining these above four lncRNAs and found that this four‐lncRNA signature could independently predict OS in HCC patients. The advantage of this study is a combination of clinical features and TCGA data to assess the survival of HCC patients by setting the lncRNA‐related risk score.

Wang et al.^31^ also identified a four‐lncRNA signature (RP11‐322E11.5, RP11‐150O12.3, AC093609.1, CTC‐297N7.9) which might be an independent prognostic biomarker for the prediction of HCC patient survival. However, compared with previous study, we used more stringent screening criteria. Firstly, we used different classification regarding the clinical information extracted from TCGA datasets. Secondly, we screened the lncRNAs which were not described in NCBI and Emsemble, the left lncRNAs were considered to have potential clinical significance for further validation. Then, the differently expressed that were 0 in more than 10% of all data were eliminated. Finally, we used “FDR <0.05 and *P *< .05” as the inclusion criteria. Therefore, the standards for bioinformatics analysis are more rigorous in our work, compared to the work in previous study. Thus, the number of candidate lncRNAs for further analyses is different in both studies. Other studies found novel biomarkers via different classification methods. Herein, it was reported in the present study that expression of four novel lncRNAs could also become a novel independent prognostic signature for HCC patients.

Accumulating evidence has presented that a series of lncRNAs could act as tumor suppressors or oncogenes in HCC. However, the roles of most lncRNAs in HCC remain largely unknown. Hu et al.^32^ found overexpressed SVUGP2 could suppress cell proliferation and suppresses the invasion ability of HCC cell lines in vitro, and tumor growth in vivo. SchLAH was found downregulated in HCC with significantly correlated with shorter overall survival of HCC patients.^33^ Moreover, HOTAIR and HOTTIP were also upregulated in HCC indicating a poorer prognosis and reduced overall survival.^34^, ^35^, ^36^

Among these above four lncRNAs in the risk score, decreased LINC00261 was identified associated with poor prognosis and metastasis in Gastric Cancer (GC).^37^ Moreover, LINC00261 was found related to cell growth, migration, cell proliferation, and cell apoptosis in endometriosis and choriocarcinoma.^38^, ^39^ Furthermore, multivariate analyses revealed that expression of CDKN2B‐AS1 could be an independent predictor for OS (*P *= .036) in GC.^40^ The other two lncRNAs (TRELM3P and GBP1P1) were not reported till now.

Moreover, we identified the genes that strongly related with these above four lncRNAs expression in HCC dataset from TCGA database. The relevant genes were mainly enriched in metabolic pathways, “Valine, leucine and isoleucine degradation,” cellular nitrogen compound metabolic process and small molecule metabolic process. However, there is no study as of yet investigated the biological and clinical function of those above four lncRNAs in HCC, there is still many research that needs to be accomplished.

These findings of the present study may have substantial clinical significance. However, the limitations should be taken into consideration in the present study. Firstly, only 1801 human lncRNAs were identified, which would be selected with a description from NCBI and Ensemble for further study. The prognostic‐related lncRNAs identified here might not represent all the lncRNAs, which were potentially related to HCC OS. Secondly, the mean time of follow‐up in the model was 813.108 days. Thus, the further study with the longer follow‐up time is warranted. Thirdly, the role of these four lncRNAs in HCC is still unknown; in vivo and in vitro experiments should be investigated in the further study.

In conclusion, by synthetically analyzing the HCC lncRNA expression profiles in TCGA database, we identified a four‐lncRNA signature, which could act as an indicator for HCC patient outcome and could be a potential independent biomarker for prognosis prediction of HCC. Future functional investigations are required to explore the mechanisms underlying the roles of these lncRNAs in HCC.

## CONFLICT OF INTEREST

The authors declared that they had no competing interests.
