# Supplementary material for: Systematic analyses of a novel lncRNA‐associated signature as the prognostic biomarker for Hepatocellular Carcinoma
Source: Cancer Med. 2018 May 15;7(7):3240–56. doi: 10.1002/cam4.1541 (PMC6051236; doi:10.1002/cam4.1541)
Supplement: Supplementary file 1 [file CAM4-7-3240-s001.docx]

**Supplementary Table 1： the differentially expressed lncRNAs in different tumor stages**

| **LncRNA** | **Differentially expressed Stages** |
| --- | --- |
| A1BG-AS1 | Ⅰ, Ⅱ, Ⅲ, Ⅳ |
| AADACP1 | Ⅰ, Ⅱ, Ⅲ, Ⅳ |
| ADAM6 | Ⅰ, Ⅱ, Ⅲ, Ⅳ |
| AFAP1-AS1 | Ⅰ, Ⅱ, Ⅲ, Ⅳ |
| AKR1C6P | Ⅰ, Ⅱ, Ⅲ, Ⅳ |
| AKR7A2P1 | Ⅰ, Ⅱ, Ⅲ, Ⅳ |
| AKR7L | Ⅰ, Ⅱ, Ⅲ, Ⅳ |
| APOC1P1 | Ⅰ, Ⅱ, Ⅲ, Ⅳ |
| AQP7P1 | Ⅰ, Ⅱ, Ⅲ, Ⅳ |
| AQP7P3 | Ⅰ, Ⅱ, Ⅲ, Ⅳ |
| B3GALT5-AS1 | Ⅰ, Ⅱ, Ⅲ, Ⅳ |
| C3P1 | Ⅰ, Ⅱ, Ⅲ, Ⅳ |
| CDC14C | Ⅰ, Ⅱ, Ⅲ, Ⅳ |
| CDKN2B-AS1 | Ⅰ, Ⅱ, Ⅲ, Ⅳ |
| CLEC4GP1 | Ⅰ, Ⅱ, Ⅲ, Ⅳ |
| CXCR2P1 | Ⅰ, Ⅱ, Ⅲ, Ⅳ |
| CYP2B7P | Ⅰ, Ⅱ, Ⅲ, Ⅳ |
| DBIL5P | Ⅰ, Ⅱ, Ⅲ, Ⅳ |
| DHRS4-AS1 | Ⅰ, Ⅱ, Ⅲ, Ⅳ |
| DLG5-AS1 | Ⅰ, Ⅱ, Ⅲ, Ⅳ |
| DNM1P35 | Ⅰ, Ⅱ, Ⅲ, Ⅳ |
| EMBP1 | Ⅰ, Ⅱ, Ⅲ, Ⅳ |
| FAM222A-AS1 | Ⅰ, Ⅱ, Ⅲ, Ⅳ |
| FAM99A | Ⅰ, Ⅱ, Ⅲ, Ⅳ |
| FAM99B | Ⅰ, Ⅱ, Ⅲ, Ⅳ |
| FOXD2-AS1 | Ⅰ, Ⅱ, Ⅲ, Ⅳ |
| GAS5 | Ⅰ, Ⅱ, Ⅲ, Ⅳ |
| GBAP1 | Ⅰ, Ⅱ, Ⅲ, Ⅳ |
| GBP1P1 | Ⅰ, Ⅱ, Ⅲ, Ⅳ |
| GLIDR | Ⅰ, Ⅱ, Ⅲ, Ⅳ |
| GOLGA2P10 | Ⅰ, Ⅱ, Ⅲ, Ⅳ |
| GOLGA2P7 | Ⅰ, Ⅱ, Ⅲ, Ⅳ |
| GUSBP11 | Ⅰ, Ⅱ, Ⅲ, Ⅳ |
| GVINP1 | Ⅰ, Ⅱ, Ⅲ, Ⅳ |
| HAND2-AS1 | Ⅰ, Ⅱ, Ⅲ, Ⅳ |
| HAR1A | Ⅰ, Ⅱ, Ⅲ, Ⅳ |
| HSPB1P1 | Ⅰ, Ⅱ, Ⅲ, Ⅳ |
| KGFLP2 | Ⅰ, Ⅱ, Ⅲ, Ⅳ |
| LINC00152 | Ⅰ, Ⅱ, Ⅲ, Ⅳ |
| LINC00176 | Ⅰ, Ⅱ, Ⅲ, Ⅳ |
| LINC00238 | Ⅰ, Ⅱ, Ⅲ, Ⅳ |
| LINC00261 | Ⅰ, Ⅱ, Ⅲ, Ⅳ |
| LINC00526 | Ⅰ, Ⅱ, Ⅲ, Ⅳ |
| LINC00685 | Ⅰ, Ⅱ, Ⅲ, Ⅳ |
| LINC00704 | Ⅰ, Ⅱ, Ⅲ, Ⅳ |
| LINC01018 | Ⅰ, Ⅱ, Ⅲ, Ⅳ |
| LINC01089 | Ⅰ, Ⅱ, Ⅲ, Ⅳ |
| LINC01554 | Ⅰ, Ⅱ, Ⅲ, Ⅳ |
| LINC01558 | Ⅰ, Ⅱ, Ⅲ, Ⅳ |
| LOC100133669 | Ⅰ, Ⅱ, Ⅲ, Ⅳ |
| LOC100240735 | Ⅰ, Ⅱ, Ⅲ, Ⅳ |
| LOC148709 | Ⅰ, Ⅱ, Ⅲ, Ⅳ |
| LOC152225 | Ⅰ, Ⅱ, Ⅲ, Ⅳ |
| LOC155060 | Ⅰ, Ⅱ, Ⅲ, Ⅳ |
| LOC283856 | Ⅰ, Ⅱ, Ⅲ, Ⅳ |
| LOC388242 | Ⅰ, Ⅱ, Ⅲ, Ⅳ |
| LOC389332 | Ⅰ, Ⅱ, Ⅲ, Ⅳ |
| LOC645166 | Ⅰ, Ⅱ, Ⅲ, Ⅳ |
| LPAL2 | Ⅰ, Ⅱ, Ⅲ, Ⅳ |
| LRRC37A6P | Ⅰ, Ⅱ, Ⅲ, Ⅳ |
| MAFG-AS1 | Ⅰ, Ⅱ, Ⅲ, Ⅳ |
| MEIS3P1 | Ⅰ, Ⅱ, Ⅲ, Ⅳ |
| MGC27382 | Ⅰ, Ⅱ, Ⅲ, Ⅳ |
| MIR4435-2HG | Ⅰ, Ⅱ, Ⅲ, Ⅳ |
| MIR99AHG | Ⅰ, Ⅱ, Ⅲ, Ⅳ |
| MSTO2P | Ⅰ, Ⅱ, Ⅲ, Ⅳ |
| MT1L | Ⅰ, Ⅱ, Ⅲ, Ⅳ |
| NAPSB | Ⅰ, Ⅱ, Ⅲ, Ⅳ |
| NCF1C | Ⅰ, Ⅱ, Ⅲ, Ⅳ |
| NUDT16P1 | Ⅰ, Ⅱ, Ⅲ, Ⅳ |
| PCAT18 | Ⅰ, Ⅱ, Ⅲ, Ⅳ |
| PLGLA | Ⅰ, Ⅱ, Ⅲ, Ⅳ |
| PTGES2-AS1 | Ⅰ, Ⅱ, Ⅲ, Ⅳ |
| PVT1 | Ⅰ, Ⅱ, Ⅲ, Ⅳ |
| SERTAD4-AS1 | Ⅰ, Ⅱ, Ⅲ, Ⅳ |
| SIGLEC16 | Ⅰ, Ⅱ, Ⅲ, Ⅳ |
| SIGLEC17P | Ⅰ, Ⅱ, Ⅲ, Ⅳ |
| SNHG1 | Ⅰ, Ⅱ, Ⅲ, Ⅳ |
| SNHG3 | Ⅰ, Ⅱ, Ⅲ, Ⅳ |
| SNHG4 | Ⅰ, Ⅱ, Ⅲ, Ⅳ |
| SSTR5-AS1 | Ⅰ, Ⅱ, Ⅲ, Ⅳ |
| TOB2P1 | Ⅰ, Ⅱ, Ⅲ, Ⅳ |
| TPTE2P1 | Ⅰ, Ⅱ, Ⅲ, Ⅳ |
| TPTEP1 | Ⅰ, Ⅱ, Ⅲ, Ⅳ |
| TREML3P | Ⅰ, Ⅱ, Ⅲ, Ⅳ |
| TSPEAR-AS2 | Ⅰ, Ⅱ, Ⅲ, Ⅳ |
| TUBA3FP | Ⅰ, Ⅱ, Ⅲ, Ⅳ |
| UCA1 | Ⅰ, Ⅱ, Ⅲ, Ⅳ |
| WDFY3-AS2 | Ⅰ, Ⅱ, Ⅲ, Ⅳ |
| ZNF252P-AS1 | Ⅰ, Ⅱ, Ⅲ, Ⅳ |
| ADGRE4P | Ⅰ, Ⅱ, Ⅲ |
| ASMTL-AS1 | Ⅰ, Ⅱ, Ⅲ |
| AURKAPS1 | Ⅰ, Ⅱ, Ⅲ |
| BEND3P3 | Ⅰ, Ⅱ, Ⅲ |
| BMS1P20 | Ⅰ, Ⅱ, Ⅲ |
| BRE-AS1 | Ⅰ, Ⅱ, Ⅲ |
| C1orf220 | Ⅰ, Ⅱ, Ⅲ |
| CCDC163P | Ⅰ, Ⅱ, Ⅲ |
| CEP83-AS1 | Ⅰ, Ⅱ, Ⅲ |
| CLRN1-AS1 | Ⅰ, Ⅱ, Ⅲ |
| CSNK1A1P1 | Ⅰ, Ⅱ, Ⅲ |
| CYP2D7 | Ⅰ, Ⅱ, Ⅲ |
| DDX12P | Ⅰ, Ⅱ, Ⅲ |
| DGCR5 | Ⅰ, Ⅱ, Ⅲ |
| DIO3OS | Ⅰ, Ⅱ, Ⅲ |
| DUSP5P1 | Ⅰ, Ⅱ, Ⅲ |
| EPB41L4A-AS2 | Ⅰ, Ⅱ, Ⅲ |
| FAM35DP | Ⅰ, Ⅱ, Ⅲ |
| FAM83A-AS1 | Ⅰ, Ⅱ, Ⅲ |
| FER1L4 | Ⅰ, Ⅱ, Ⅲ |
| FIRRE | Ⅰ, Ⅱ, Ⅲ |
| FLJ23867 | Ⅰ, Ⅱ, Ⅲ |
| FLVCR1-AS1 | Ⅰ, Ⅱ, Ⅲ |
| GGT3P | Ⅰ, Ⅱ, Ⅲ |
| GK3P | Ⅰ, Ⅱ, Ⅲ |
| GUSBP5 | Ⅰ, Ⅱ, Ⅲ |
| H19 | Ⅰ, Ⅱ, Ⅲ |
| HCG11 | Ⅰ, Ⅱ, Ⅲ |
| HCG4 | Ⅰ, Ⅱ, Ⅲ |
| ID2B | Ⅰ, Ⅱ, Ⅲ |
| IGF2-AS | Ⅰ, Ⅱ, Ⅲ |
| LINC00092 | Ⅰ, Ⅱ, Ⅲ |
| LINC00310 | Ⅰ, Ⅱ, Ⅲ |
| LINC00574 | Ⅰ, Ⅱ, Ⅲ |
| LINC00598 | Ⅰ, Ⅱ, Ⅲ |
| LINC00893 | Ⅰ, Ⅱ, Ⅲ |
| LINC00894 | Ⅰ, Ⅱ, Ⅲ |
| LINC00896 | Ⅰ, Ⅱ, Ⅲ |
| LINC00924 | Ⅰ, Ⅱ, Ⅲ |
| LINC01126 | Ⅰ, Ⅱ, Ⅲ |
| LINC01139 | Ⅰ, Ⅱ, Ⅲ |
| LINC01140 | Ⅰ, Ⅱ, Ⅲ |
| LINC01512 | Ⅰ, Ⅱ, Ⅲ |
| LINC01550 | Ⅰ, Ⅱ, Ⅲ |
| LOC100132111 | Ⅰ, Ⅱ, Ⅲ |
| LOC100190940 | Ⅰ, Ⅱ, Ⅲ |
| LOC100270804 | Ⅰ, Ⅱ, Ⅲ |
| LOC153684 | Ⅰ, Ⅱ, Ⅲ |
| LOC400927 | Ⅰ, Ⅱ, Ⅲ |
| LOC728743 | Ⅰ, Ⅱ, Ⅲ |
| MBL1P | Ⅰ, Ⅱ, Ⅲ |
| MCM3AP-AS1 | Ⅰ, Ⅱ, Ⅲ |
| MIR503HG | Ⅰ, Ⅱ, Ⅲ |
| MIR924HG | Ⅰ, Ⅱ, Ⅲ |
| MT1DP | Ⅰ, Ⅱ, Ⅲ |
| NCF1B | Ⅰ, Ⅱ, Ⅲ |
| NSUN5P1 | Ⅰ, Ⅱ, Ⅲ |
| NSUN5P2 | Ⅰ, Ⅱ, Ⅲ |
| NUDT9P1 | Ⅰ, Ⅱ, Ⅲ |
| PMS2P3 | Ⅰ, Ⅱ, Ⅲ |
| PPP4R3CP | Ⅰ, Ⅱ, Ⅲ |
| PRR26 | Ⅰ, Ⅱ, Ⅲ |
| SFTA1P | Ⅰ, Ⅱ, Ⅲ |
| SMIM10L2A | Ⅰ, Ⅱ, Ⅲ |
| SNHG12 | Ⅰ, Ⅱ, Ⅲ |
| SNORA71E | Ⅰ, Ⅱ, Ⅲ |
| SNX29P2 | Ⅰ, Ⅱ, Ⅲ |
| TCAM1P | Ⅰ, Ⅱ, Ⅲ |
| UCKL1-AS1 | Ⅰ, Ⅱ, Ⅲ |
| UNQ6494 | Ⅰ, Ⅱ, Ⅲ |
| LOC653653 | Ⅰ, Ⅱ, Ⅳ |
| ZMIZ1-AS1 | Ⅰ, Ⅲ, Ⅳ |
| ANXA2P1 | Ⅱ, Ⅲ, Ⅳ |
| CCL15-CCL14 | Ⅱ, Ⅲ, Ⅳ |
| CES1P1 | Ⅱ, Ⅲ, Ⅳ |
| MIR22HG | Ⅱ, Ⅲ, Ⅳ |
| RPLP0P2 | Ⅱ, Ⅲ, Ⅳ |
| SNHG7 | Ⅱ, Ⅲ, Ⅳ |
| FAM223B | Ⅰ, Ⅱ |
| FLJ12825 | Ⅰ, Ⅱ |
| HCG27 | Ⅰ, Ⅱ |
| INE2 | Ⅰ, Ⅱ |
| LINC00482 | Ⅰ, Ⅱ |
| LINC01226 | Ⅰ, Ⅱ |
| DSCR8 | Ⅰ, Ⅲ |
| HEXA-AS1 | Ⅰ, Ⅲ |
| HLA-DRB6 | Ⅰ, Ⅲ |
| NPSR1-AS1 | Ⅰ, Ⅲ |
| RPPH1 | Ⅰ, Ⅲ |
| HNF1A-AS1 | Ⅰ, Ⅳ |
| LINC00950 | Ⅰ, Ⅳ |
| LINC01341 | Ⅰ, Ⅳ |
| A2M-AS1 | Ⅱ, Ⅲ |
| ABCC6P1 | Ⅱ, Ⅲ |
| AOC4P | Ⅱ, Ⅲ |
| CECR7 | Ⅱ, Ⅲ |
| CPS1-IT1 | Ⅱ, Ⅲ |
| DKFZp779M0652 | Ⅱ, Ⅲ |
| DNMBP-AS1 | Ⅱ, Ⅲ |
| DPY19L2P2 | Ⅱ, Ⅲ |
| DRAIC | Ⅱ, Ⅲ |
| FAM35BP | Ⅱ, Ⅲ |
| FAR2P1 | Ⅱ, Ⅲ |
| FBXL19-AS1 | Ⅱ, Ⅲ |
| GABARAPL3 | Ⅱ, Ⅲ |
| GUCY1B2 | Ⅱ, Ⅲ |
| LINC00242 | Ⅱ, Ⅲ |
| LINC00488 | Ⅱ, Ⅲ |
| LINC00628 | Ⅱ, Ⅲ |
| LINC00634 | Ⅱ, Ⅲ |
| LINC01101 | Ⅱ, Ⅲ |
| LOC146880 | Ⅱ, Ⅲ |
| LOC153910 | Ⅱ, Ⅲ |
| LOC399815 | Ⅱ, Ⅲ |
| LOC441666 | Ⅱ, Ⅲ |
| LOC642846 | Ⅱ, Ⅲ |
| LOC642852 | Ⅱ, Ⅲ |
| MIR100HG | Ⅱ, Ⅲ |
| MIR600HG | Ⅱ, Ⅲ |
| NFE2L3P2 | Ⅱ, Ⅲ |
| PART1 | Ⅱ, Ⅲ |
| PTTG3P | Ⅱ, Ⅲ |
| SLC6A10P | Ⅱ, Ⅲ |
| SPATA41 | Ⅱ, Ⅲ |
| SUZ12P1 | Ⅱ, Ⅲ |
| TMPO-AS1 | Ⅱ, Ⅲ |
| ABCA17P | Ⅱ, Ⅳ |
| LOC285629 | Ⅱ, Ⅳ |
| TPRXL | Ⅱ, Ⅳ |
| ADORA2A-AS1 | Ⅲ, Ⅳ |
| ALOX12P2 | Ⅲ, Ⅳ |
| ANXA2P2 | Ⅲ, Ⅳ |
| CMAHP | Ⅲ, Ⅳ |
| LINC01091 | Ⅲ, Ⅳ |
| LOC100133985 | Ⅲ, Ⅳ |
| LOC401127 | Ⅲ, Ⅳ |
| TCL6 | Ⅲ, Ⅳ |
| HULC | Ⅰ |
| IGF2BP2-AS1 | Ⅰ |
| LINC00926 | Ⅰ |
| PRKY | Ⅰ |
| ZNF204P | Ⅰ |
| ALMS1P1 | Ⅱ |
| CCDC144B | Ⅱ |
| HOXA11-AS | Ⅱ |
| KRT17P5 | Ⅱ |
| LINC01128 | Ⅱ |
| LOC553137 | Ⅱ |
| MRPL23-AS1 | Ⅱ |
| PWARSN | Ⅱ |
| RNF126P1 | Ⅱ |
| THUMPD3-AS1 | Ⅱ |
| WEE2-AS1 | Ⅱ |
| ZFHX4-AS1 | Ⅱ |
| ANKRD18DP | Ⅲ |
| DGCR9 | Ⅲ |
| FAM182B | Ⅲ |
| FAM66D | Ⅲ |
| GTSE1-AS1 | Ⅲ |
| HERC2P2 | Ⅲ |
| HSP90AB2P | Ⅲ |
| KIAA0125 | Ⅲ |
| LINC00887 | Ⅲ |
| LINC01622 | Ⅲ |
| LOC100126784 | Ⅲ |
| LOC256880 | Ⅲ |
| LOC440173 | Ⅲ |
| RPL13P5 | Ⅲ |
| RPL34-AS1 | Ⅲ |
| SUGT1P3 | Ⅲ |
| ZNF192P1 | Ⅲ |
| ZNF300P1 | Ⅲ |
| ZNF767P | Ⅲ |
| ZNF826P | Ⅲ |
| ABCC6P2 | Ⅳ |
| AGAP7P | Ⅳ |
| AKR1C8P | Ⅳ |
| ANKRD36BP2 | Ⅳ |
| ANXA2P3 | Ⅳ |
| ATP5EP2 | Ⅳ |
| C7orf13 | Ⅳ |
| CASC2 | Ⅳ |
| CLUHP3 | Ⅳ |
| CTAGE10P | Ⅳ |
| CYP1B1-AS1 | Ⅳ |
| DCAF13P3 | Ⅳ |
| FAM86B3P | Ⅳ |
| FAM86JP | Ⅳ |
| FAM95B1 | Ⅳ |
| GNAS-AS1 | Ⅳ |
| LHFPL3-AS2 | Ⅳ |
| LINC00174 | Ⅳ |
| LINC00239 | Ⅳ |
| LINC00240 | Ⅳ |
| LINC00671 | Ⅳ |
| LINC00839 | Ⅳ |
| LINC00982 | Ⅳ |
| LINC01011 | Ⅳ |
| LINC01134 | Ⅳ |
| LOC100268168 | Ⅳ |
| LOC143188 | Ⅳ |
| LOC284023 | Ⅳ |
| LOC606724 | Ⅳ |
| LOC646471 | Ⅳ |
| LOC727896 | Ⅳ |
| LOH12CR2 | Ⅳ |
| MGC16275 | Ⅳ |
| MIR4697HG | Ⅳ |
| MSL3P1 | Ⅳ |
| MST1P2 | Ⅳ |
| NDUFB2-AS1 | Ⅳ |
| NME2P1 | Ⅳ |
| PMS2CL | Ⅳ |
| POM121L10P | Ⅳ |
| PP7080 | Ⅳ |
| PSORS1C3 | Ⅳ |
| RPL13AP20 | Ⅳ |
| RPSAP58 | Ⅳ |
| RRN3P1 | Ⅳ |
| SNHG11 | Ⅳ |
| SNHG17 | Ⅳ |
| TDGF1P3 | Ⅳ |
| TTC41P | Ⅳ |
| USP27X-AS1 | Ⅳ |
| WT1-AS | Ⅳ |
| ZEB1-AS1 | Ⅳ |
| ZNF788 | Ⅳ |
