# Supplementary material for: Systematic analyses of a novel lncRNA‐associated signature as the prognostic biomarker for Hepatocellular Carcinoma
Source: Cancer Med. 2018 May 15;7(7):3240–56. doi: 10.1002/cam4.1541 (PMC6051236; doi:10.1002/cam4.1541)
Supplement: Supplementary file 2 [file CAM4-7-3240-s002.doc]

**Supplementary Table 2: The co-expressed mRNAs of the four lncRNAs (LINC00261, TRELM3P, GBP1P1, and CDKN2B-AS1).**

| lncRNA | Co-expressed mRNA | Pearson |R| |
| --- | --- | --- |
| LINC00261  LINC00261  LINC00261  LINC00261  LINC00261  LINC00261  LINC00261  LINC00261  LINC00261  LINC00261  LINC00261  LINC00261  LINC00261  LINC00261  LINC00261  LINC00261  LINC00261  LINC00261  LINC00261  LINC00261  LINC00261  LINC00261  LINC00261  LINC00261  LINC00261  LINC00261  LINC00261  LINC00261  LINC00261  LINC00261  LINC00261  LINC00261  LINC00261  LINC00261  LINC00261  LINC00261  LINC00261  LINC00261  LINC00261  LINC00261  LINC00261  LINC00261  LINC00261  LINC00261  LINC00261  LINC00261  LINC00261  LINC00261  LINC00261  LINC00261  LINC00261  LINC00261  LINC00261  LINC00261  LINC00261  LINC00261  LINC00261  LINC00261  LINC00261  LINC00261  LINC00261  LINC00261  LINC00261  LINC00261  LINC00261  LINC00261  LINC00261  LINC00261  LINC00261  LINC00261  LINC00261  LINC00261  LINC00261  LINC00261  LINC00261  LINC00261  LINC00261  LINC00261  LINC00261  LINC00261  LINC00261  LINC00261  LINC00261  LINC00261  LINC00261  LINC00261  LINC00261  LINC00261  LINC00261  LINC00261  LINC00261  LINC00261  LINC00261  LINC00261  LINC00261  LINC00261  LINC00261  LINC00261  LINC00261  LINC00261  LINC00261  LINC00261  LINC00261  LINC00261  LINC00261  LINC00261  LINC00261  LINC00261  LINC00261  LINC00261  LINC00261  LINC00261  LINC00261  LINC00261  LINC00261  LINC00261  LINC00261  LINC00261  LINC00261  LINC00261  LINC00261  LINC00261  LINC00261  LINC00261  LINC00261  LINC00261  LINC00261  LINC00261  LINC00261  LINC00261  LINC00261  LINC00261  LINC00261  LINC00261  LINC00261  LINC00261  LINC00261  LINC00261  LINC00261  LINC00261  LINC00261  LINC00261  LINC00261  LINC00261  LINC00261  LINC00261  LINC00261  LINC00261  LINC00261  LINC00261  LINC00261  LINC00261  LINC00261  LINC00261  LINC00261  LINC00261  LINC00261  LINC00261  LINC00261  LINC00261  LINC00261  LINC00261  LINC00261  LINC00261  LINC00261  LINC00261  LINC00261  LINC00261  LINC00261  LINC00261  LINC00261  LINC00261  LINC00261  LINC00261  LINC00261  LINC00261  LINC00261  LINC00261  LINC00261  LINC00261  LINC00261  LINC00261  LINC00261  LINC00261  LINC00261  LINC00261  LINC00261  LINC00261  LINC00261  LINC00261  LINC00261  LINC00261  LINC00261  LINC00261  LINC00261  LINC00261  LINC00261  LINC00261  LINC00261  LINC00261  LINC00261  LINC00261  LINC00261  LINC00261  LINC00261  LINC00261  LINC00261  LINC00261  LINC00261  LINC00261  LINC00261  LINC00261  LINC00261  LINC00261  LINC00261  LINC00261  LINC00261  LINC00261  LINC00261  LINC00261  LINC00261  LINC00261  LINC00261  LINC00261  LINC00261  LINC00261  LINC00261  LINC00261  LINC00261  LINC00261  LINC00261  LINC00261  LINC00261  LINC00261  LINC00261  LINC00261  LINC00261  LINC00261  LINC00261  LINC00261  LINC00261  LINC00261  LINC00261  LINC00261  LINC00261  LINC00261  LINC00261  LINC00261  LINC00261  LINC00261  LINC00261  LINC00261  LINC00261  LINC00261  LINC00261  LINC00261  LINC00261  LINC00261  LINC00261  LINC00261  LINC00261  LINC00261  LINC00261  LINC00261  LINC00261  LINC00261  LINC00261  LINC00261  LINC00261  LINC00261  LINC00261  LINC00261  LINC00261  LINC00261  LINC00261  LINC00261  LINC00261  LINC00261  LINC00261  LINC00261  LINC00261  LINC00261  LINC00261  LINC00261  LINC00261  LINC00261  LINC00261  LINC00261  LINC00261  LINC00261  LINC00261  LINC00261  LINC00261  LINC00261  LINC00261  LINC00261  LINC00261  LINC00261  LINC00261  LINC00261  LINC00261  LINC00261  LINC00261  LINC00261  LINC00261  LINC00261  LINC00261  LINC00261  LINC00261  LINC00261  LINC00261  LINC00261  LINC00261  LINC00261  LINC00261  LINC00261  LINC00261  LINC00261  LINC00261  LINC00261  LINC00261  LINC00261  LINC00261  LINC00261  LINC00261  LINC00261  LINC00261  LINC00261  LINC00261  LINC00261  LINC00261  LINC00261  LINC00261  LINC00261  LINC00261  LINC00261  LINC00261  LINC00261  LINC00261  LINC00261  LINC00261  LINC00261  LINC00261  LINC00261  LINC00261  LINC00261  LINC00261  LINC00261  LINC00261  LINC00261  LINC00261  LINC00261  LINC00261  LINC00261  LINC00261  LINC00261  LINC00261  LINC00261  LINC00261  LINC00261  LINC00261  LINC00261  LINC00261  LINC00261  LINC00261  LINC00261  LINC00261  LINC00261  LINC00261  LINC00261  LINC00261  LINC00261  LINC00261  LINC00261  LINC00261  LINC00261  LINC00261  LINC00261  LINC00261  LINC00261  LINC00261  LINC00261  LINC00261  LINC00261  LINC00261  LINC00261  LINC00261  LINC00261  LINC00261  LINC00261  LINC00261  LINC00261  LINC00261  LINC00261  LINC00261  LINC00261  LINC00261  LINC00261  LINC00261  LINC00261  LINC00261  LINC00261  LINC00261  LINC00261  LINC00261  LINC00261  LINC00261  LINC00261  LINC00261  LINC00261  LINC00261  LINC00261  LINC00261  LINC00261  LINC00261  LINC00261  LINC00261  LINC00261  LINC00261  LINC00261  LINC00261  LINC00261  LINC00261  LINC00261  LINC00261  LINC00261  LINC00261  TREML3P  TREML3P  TREML3P  TREML3P  TREML3P  TREML3P  TREML3P  TREML3P  TREML3P  TREML3P  TREML3P  TREML3P  TREML3P  TREML3P  TREML3P  TREML3P  TREML3P  TREML3P  TREML3P  TREML3P  TREML3P  TREML3P  TREML3P  TREML3P  TREML3P  TREML3P  TREML3P  TREML3P  TREML3P  TREML3P  TREML3P  TREML3P  TREML3P  TREML3P  TREML3P  TREML3P  GBP1P1  GBP1P1  GBP1P1  GBP1P1  GBP1P1  GBP1P1  GBP1P1  GBP1P1  GBP1P1  GBP1P1  GBP1P1  GBP1P1  GBP1P1  GBP1P1  GBP1P1  GBP1P1  GBP1P1  GBP1P1  GBP1P1  GBP1P1  GBP1P1  GBP1P1  GBP1P1  GBP1P1  GBP1P1  GBP1P1  GBP1P1  GBP1P1  GBP1P1  GBP1P1  GBP1P1  GBP1P1  GBP1P1  GBP1P1  GBP1P1  GBP1P1  GBP1P1  GBP1P1  GBP1P1  GBP1P1  GBP1P1  GBP1P1  GBP1P1  GBP1P1  GBP1P1  GBP1P1  GBP1P1  GBP1P1  GBP1P1  GBP1P1  GBP1P1  GBP1P1  GBP1P1  GBP1P1  GBP1P1  GBP1P1  GBP1P1  GBP1P1  GBP1P1  GBP1P1  GBP1P1  GBP1P1  GBP1P1  GBP1P1  GBP1P1  GBP1P1  GBP1P1  GBP1P1  GBP1P1  GBP1P1  GBP1P1  GBP1P1  GBP1P1  GBP1P1  GBP1P1  GBP1P1  GBP1P1  GBP1P1  GBP1P1  GBP1P1  GBP1P1  GBP1P1  GBP1P1  GBP1P1  GBP1P1  GBP1P1  GBP1P1  GBP1P1  GBP1P1  GBP1P1  GBP1P1  GBP1P1  GBP1P1  GBP1P1  GBP1P1  GBP1P1  GBP1P1  GBP1P1  GBP1P1  GBP1P1  GBP1P1  GBP1P1  GBP1P1  GBP1P1  GBP1P1  GBP1P1  GBP1P1  GBP1P1  GBP1P1  GBP1P1  GBP1P1  GBP1P1  GBP1P1  GBP1P1  GBP1P1  GBP1P1  GBP1P1  GBP1P1  GBP1P1  GBP1P1  GBP1P1  GBP1P1  GBP1P1  GBP1P1  GBP1P1  GBP1P1  GBP1P1  GBP1P1  GBP1P1  GBP1P1  GBP1P1  GBP1P1  CDKN2B-AS1  CDKN2B-AS1  CDKN2B-AS1  CDKN2B-AS1  CDKN2B-AS1  CDKN2B-AS1  CDKN2B-AS1  CDKN2B-AS1  CDKN2B-AS1  CDKN2B-AS1  CDKN2B-AS1  CDKN2B-AS1  CDKN2B-AS1  CDKN2B-AS1  CDKN2B-AS1  CDKN2B-AS1  CDKN2B-AS1  CDKN2B-AS1  CDKN2B-AS1  CDKN2B-AS1  CDKN2B-AS1  CDKN2B-AS1  CDKN2B-AS1  CDKN2B-AS1  CDKN2B-AS1  CDKN2B-AS1  CDKN2B-AS1  CDKN2B-AS1  CDKN2B-AS1  CDKN2B-AS1  CDKN2B-AS1 | NAT1  NAT2  ABAT  ACAA1  ACACB  ACADL  ACADM  ACADS  ACADSB  ACAT1  ACOX1  ADH1A  ADH1B  ADH4  ADH6  ADK  ADRA1A  AFM  AGL  AGXT  ALAD  ALAS1  ALB  ALDH2  ALDOB  AOX1  APOF  APOB  ABCC6  RHOB  RND3  ARSD  AZGP1  BAK1  BCKDHB  BDH1  BHMT  KLF9  SERPING1  C1R  C1S  C6  C8A  C8B  SLC25A20  CAT  CBS  CDO1  CFL2  SLC31A1  CP  CPB2  CPN2  CPT2  CRHBP  CTBS  CTSO  CYB5A  CYP1A2  CYP2C19  CYP2C8  CYP2C9  CYP4A11  CYP8B1  DBT  DMD  SARDH  DNASE1L3  DPYS  RCAN1  DUSP1  ECHS1  EHHADH  EIF5  EPHX2  ESR1  ETFDH  ETS2  F9  F11  ACSL1  FBP1  FOXO1  FMO3  FXN  GATM  GC  GCH1  GCKR  GDF2  GHR  GNAO1  GPD1  GSTZ1  GYS2  HMGCL  HMGCS2  HPX  ID2  CFI  IGF1  IGFALS  ILF2  INHBC  ITIH4  IVD  KCNN2  KLKB1  LCAT  LDLR  LMAN1  LPA  CYP4F3  MAOB  MAT1A  MBL2  MCC  CTAGE5  ALDH6A1  MYO1B  MSRA  MTHFD1  MTTP  MUT  MYD88  MYLK  NFIA  NFIL3  NTF3  OTC  PEBP1  PCSK6  PAH  PCK1  PCK2  PEX13  PGM1  PIK3R1  PLG  PLGLB2  SERPINF2  PON1  PON3  MAP2K1  PROS1  MASP1  PTPN3  PZP  QDPR  RBL2  RBP4  RNASE4  RORA  SAA4  ACSM3  SALL1  SCP2  SDHD  SEPP1  ST6GAL1  SLC2A2  SLC7A2  SLC10A1  SLC16A2  SLC22A1  SLC22A3  SORD  SORL1  SRD5A1  SRD5A2  AKR1D1  SUOX  TCOF1  TMBIM6  SEC62  TPMT  TSPYL1  UGT2B7  UGT2B10  VIPR1  XDH  PTP4A1  ALDH5A1  EPM2A  NRIP1  ACOX2  BBOX1  PEX3  CYP4F2  KMO  RDH16  PLPP3  HSD17B6  SNX4  MPDZ  SUCLG2  SOCS2  NR1I2  PROZ  RGN  SLC28A1  REPS2  LRAT  SOCS6  GRHPR  EI24  EDEM1  MFAP3L  FARP2  KBTBD11  CD302  NR1H4  GNE  ABCC9  FAM13A  RCL1  DDX39A  STX6  GLYAT  SMYD5  ABCA8  PEMT  ST3GAL6  SEC24B  MCRS1  ACAA2  DCTN2  COLEC10  SLCO1B1  PDLIM5  ERLIN1  FAM189B  IQGAP2  FTCD  CLPX  PGRMC1  EHMT2  PRDX3  FERMT2  SLC27A5  SLC27A2  ABHD2  ADAMTS13  ANXA10  PROSC  DUSP10  CHP1  COBLL1  CPEB3  HMGXB3  VWA8  SLC35D1  KIAA0922  DNAJC16  SYNE1  MLYCD  RYBP  ABCA6  SLC39A14  GABARAPL1  SHPK  BHMT2  METTL7A  FAM149A  ARHGEF26  HIBCH  CIDEB  NAAA  MMADHC  ANGPTL3  NKIRAS1  GIT1  C11orf54  UBE2T  DMGDH  NRBF2  PIK3R4  ZBTB21  AK3  FAHD2A  ISOC1  SEPSECS  ABHD5  SERPINA10  AADAT  TUBE1  HAO2  CLEC1B  PIPOX  C1RL  CYP39A1  CSAD  PRKAG2  SNX9  PCYOX1  MPC1  UPB1  TM6SF2  PANK1  RIPK4  C21orf91  HAO1  SLC38A2  FAM134B  EPB41L4B  GRAMD1C  GIPC2  SNRK  RNF125  ACSM5  MARC2  PID1  SLC38A4  BSDC1  LARP1B  STEAP3  CCDC25  ECHDC2  PI4K2B  LIN7C  DHTKD1  PIGV  CDC37L1  C8orf4  PLSCR4  PDSS2  TMEM27  ABHD6  NDRG2  SERINC1  GBA3  PBLD  MCCC2  ALDH8A1  CSRNP1  RMND5A  CYP3A43  ELOVL1  GNPNAT1  AGXT2  TRPM8  C2orf47  TANGO6  PPP1R3B  ZNF668  KDM8  THNSL1  CPED1  MOGAT2  KLHL15  DCAF11  STARD5  COL18A1  DUSP16  APOL6  ITCH  LONP2  ANGPTL6  BCO2  SLC41A2  ACAD11  EVA1A  ZMYND12  USP38  MCEE  CNDP1  CBR4  ADGRG7  MFSD2A  PHLDB2  N4BP2L1  CERS5  DEPDC7  GLYATL1  SFXN1  HOGA1  LARP4  PALM2  GPR146  FAM122A  APOA5  ACSM2A  DCUN1D3  ACVR1C  UROC1  COL6A6  SLC9B2  NADK2  CMBL  ACOT12  TTC36  LYRM5  TMEM56  PUS10  RMDN2  ZFP1  ADGRA3  MMAA  OIT3  LDHD  C1orf168  SMIM14  TMEM192  TAPT1  OAF  RNF152  CES5A  NT5DC1  ETFBKMT  SERPINA11  SPRYD4  SLC46A3  SLC25A47  ZADH2  CYP4V2  PRR18  MICU3  ATP11C  HSD17B13  TMEM220  IYD  AGMO  IDNK  CDNF  RNF165  DNAJC25  INS-IGF2  CCR3  F2RL2  GIP  GLA  MATN3  OPRK1  PPP2R2C  TNP1  NPHS2  KRT37  MMP20  TTLL4  HS3ST4  PSMD14  SPAG11B  CPLX2  HECW1  SYNGR4  CABYR  SACS  P2RY10  TREM1  C2orf83  C8orf34  DEFB104A  GABRR3  SLC10A4  DEFB116  TREML4  MAGEB18  ZAR1  SULT6B1  C8orf22  SPAG11A  CT45A2  FRG2C  FASLG  B2M  CASP1  RUNX3  CD2  CD3E  CD3G  CD247  CD8A  CD8B  CD27  CD53  CD74  CCR5  CTLA4  CTSW  DOCK2  GBP1  CXCR3  GPR18  GZMA  NCKAP1L  HLA-DMB  HLA-DOA  HLA-DOB  HLA-DPA1  HLA-DPB1  HLA-DQB1  HLA-DRA  HLA-E  IFIT3  IFNG  IL12RB1  IL16  IDO1  CXCL10  IRF1  ITK  KLRC3  KLRD1  LCK  LCP2  SH2D1A  CIITA  CXCL9  OAS3  PRF1  PSMB9  PTPN7  PTPRC  RAC2  CCL5  CXCL11  TRIM21  STAT1  TAP1  TAP2  WARS  WAS  ZAP70  EOMES  GPR65  NMI  UBE2L6  LPXN  AKAP5  ACAP1  ARHGAP25  RASGRP1  CD96  TRIM22  BTN3A3  TRAFD1  CORO1A  MAP4K1  SP140  TFEC  KLRK1  IPCEF1  PTPN22  DAPP1  SIT1  ICOS  GPR171  TBX21  IL21R  TRAT1  SLC15A3  BIN2  ETV7  CECR1  UBASH3A  SASH3  PARP14  SAMD9  GIMAP4  SIRPG  SLAMF7  APOBEC3G  MYO1G  PARVG  IFIH1  ARHGAP9  PVRIG  ZBED2  TRAF3IP3  ZBP1  NLRC5  SLA2  GPR174  SYTL3  EPSTI1  SLAMF6  FCRL3  GBP4  GBP5  BATF2  SNX20  ZNF831  HAPLN3  PYHIN1  BTLA  JAKMIP1  SAMD3  TTC24  PATL2  TIGIT  SAMD9L  ARHGAP30  THEMIS  DTHD1  FAM26F  CDKN1C  CDKN2A  CDKN2B  DTYMK  FUT7  H2AFZ  MCM2  MCM7  PPY  PYY  RFC4  PHLDA2  XRCC3  HIST1H3C  HIST1H3I  CRLF1  KNTC1  TROAP  TRAIP  POLQ  CEP131  TMEM59L  TIPIN  CENPM  C16orf59  ARHGEF39  ARHGAP33  RMI2  BPIFA2  RDM1  GPRC6A | 0.561720267  0.698696647  0.673167766  0.64785319  0.563305546  0.573618902  0.622852432  0.55965163  0.646513477  0.621785874  0.630931352  0.643251909  0.609250044  0.715376976  0.656478231  0.563199385  0.671684568  0.601483991  0.582129665  0.52995105  0.562918285  0.568947605  0.535736298  0.687001892  0.570233586  0.571049914  0.563778316  0.574450722  0.558229267  0.57164258  0.511085914  0.533047862  0.591991928  0.506360985  0.711591123  0.531425203  0.562292091  0.589414873  0.572437989  0.562761046  0.638072295  0.646463798  0.681374006  0.618004911  0.577583336  0.706919169  0.522619152  0.622924331  0.547069513  0.714797237  0.546678611  0.648681356  0.569395655  0.546468341  0.519977444  0.573348084  0.532560009  0.572576851  0.575987548  0.557717689  0.661942986  0.612266382  0.632160295  0.605903885  0.635654005  0.576833781  0.619420407  0.506310148  0.590060468  0.600157314  0.511203744  0.570440044  0.694054903  0.523584073  0.66212293  0.679111396  0.686339418  0.52184165  0.689076297  0.610547048  0.705031852  0.608367399  0.669893053  0.538542919  0.548019982  0.593983809  0.601935147  0.507539118  0.502465408  0.506737947  0.718474208  0.57021341  0.589848397  0.601014943  0.728756925  0.511278771  0.51638812  0.589590323  0.60776494  0.593908798  0.637787487  0.634933689  0.518493161  0.506066811  0.532773351  0.672712354  0.594431318  0.634356788  0.653248077  0.503409494  0.504965424  0.51394913  0.5196829  0.565507985  0.700811843  0.591970534  0.517962169  0.548978426  0.646380496  0.623152131  0.514940566  0.567936578  0.563866522  0.63152488  0.584559996  0.511094725  0.579529817  0.559138918  0.51480146  0.521488578  0.544184278  0.530014258  0.589977519  0.632374812  0.566134364  0.662460935  0.609699817  0.58904131  0.699695099  0.608922425  0.500612729  0.586710221  0.55339474  0.670537076  0.578454578  0.657595524  0.51776855  0.53675709  0.516819998  0.528380113  0.562166607  0.508160214  0.569620232  0.544545617  0.679600388  0.645384494  0.698042737  0.57337939  0.555132091  0.535310702  0.664235747  0.509728741  0.550212828  0.584837609  0.625024058  0.51784628  0.558734237  0.60620248  0.57406321  0.590277599  0.64896012  0.601464514  0.537101442  0.636836141  0.614041463  0.508444985  0.568004729  0.64804177  0.586281714  0.565990753  0.514797332  0.544112768  0.521061883  0.511172784  0.540171933  0.633886865  0.506959982  0.523350976  0.623234366  0.518416217  0.675958505  0.62835557  0.675859256  0.519807165  0.554945882  0.531586077  0.593449359  0.603711232  0.599628309  0.559410854  0.510347263  0.522498658  0.581571175  0.58120211  0.528468261  0.523688513  0.576719722  0.670914112  0.535721368  0.557072594  0.658764817  0.558454383  0.654274316  0.603234978  0.538290814  0.590244485  0.509786215  0.522878935  0.637126793  0.527594427  0.532806954  0.514050163  0.652382265  0.523599854  0.534564045  0.604374596  0.50292536  0.564727026  0.602285298  0.589265264  0.616285006  0.504624054  0.588932658  0.552446914  0.5676432  0.562447314  0.536579661  0.559591111  0.50805918  0.530464414  0.565377349  0.55004611  0.523709734  0.570868633  0.540379919  0.538775564  0.579319261  0.618660986  0.704085742  0.513394952  0.56092976  0.671399359  0.501046231  0.52812653  0.50195173  0.596380191  0.508892231  0.514124132  0.549541212  0.533393224  0.542954102  0.523380016  0.688143976  0.551718268  0.576537637  0.558556397  0.702583464  0.592977985  0.519641693  0.566812321  0.566829349  0.528461706  0.643848297  0.500896029  0.701115178  0.569043711  0.504370998  0.507811887  0.539067643  0.535423619  0.5813944  0.62617868  0.51297475  0.594646316  0.716262218  0.658244608  0.612803215  0.502045615  0.590968418  0.691608352  0.659807453  0.527303719  0.559879273  0.503315964  0.528717761  0.561206794  0.532551312  0.538483129  0.69983048  0.509296879  0.617337711  0.633658355  0.549333912  0.58571992  0.68998857  0.567047078  0.581098197  0.556421555  0.567342652  0.649463325  0.63305372  0.516207879  0.616199382  0.53031751  0.628147851  0.599629672  0.501311621  0.505539263  0.546407025  0.628778272  0.578022758  0.52901922  0.57005868  0.577391713  0.631889795  0.537926285  0.550968236  0.574393524  0.590939784  0.549984973  0.688765539  0.679028737  0.551887975  0.692426254  0.53328376  0.501210221  0.520457393  0.522858297  0.556865372  0.525258881  0.520469199  0.503272351  0.510668554  0.517190607  0.528473985  0.663746522  0.507552444  0.673855189  0.567934433  0.642797665  0.506767891  0.621799698  0.555524938  0.525113078  0.502722312  0.564026576  0.569878468  0.590546362  0.584856282  0.628027023  0.743009606  0.527426084  0.503677809  0.556330327  0.519543385  0.677280624  0.650550902  0.523748345  0.565946322  0.547094593  0.656944007  0.530701706  0.628918727  0.769338454  0.602030698  0.593697792  0.584275783  0.574166909  0.579890403  0.524321232  0.526454634  0.602133689  0.518514508  0.540393429  0.634698601  0.504793455  0.590809252  0.593269567  0.502769784  0.586113789  0.571931006  0.643860776  0.688147191  0.620685196  0.603883515  0.548997285  0.522848697  0.604070954  0.511170937  0.546158575  0.56230876  0.562703722  0.559055981  0.635369753  0.512908566  0.600412636  0.529327202  0.565268222  0.598373306  0.564908942  0.617092579  0.521886652  0.595666929  0.530053154  0.674434344  0.521385816  0.525470671  0.617680943  0.613368522  0.592139716  0.659397491  0.534066141  0.510587436  0.503956163  0.59885649  0.638220091  0.595066818  0.709263402  0.606152  0.61814639  0.538018491  0.600732764  0.543508749  0.525099152  0.876592475  0.506184251  0.715199117  0.873606218  0.570948472  0.589763805  0.514657682  0.553759793  0.552273644  0.607802111  0.622176529  0.635106018  0.574725966  0.621398375  0.649961182  0.512762588  0.731894368  0.874944208  0.568535939  0.743796272  0.874944208  0.834328252  0.556983778  0.597348968  0.625932927  0.614560292  0.791505394  0.598457686  0.768867598  0.595949413  0.587703782  0.630662492  0.501709016  0.541174949  0.553392765  0.578178795  0.51729792  0.665525876  0.57036206  0.515927559  0.535920876  0.564028505  0.575060863  0.54511338  0.531916806  0.509061588  0.649553915  0.561924044  0.524457095  0.526272214  0.523308745  0.617524734  0.599725121  0.614997581  0.574740684  0.537394862  0.520176822  0.590810887  0.539518807  0.540293219  0.595451155  0.631426496  0.500904067  0.590136502  0.605140283  0.601794835  0.508148106  0.557932529  0.521778608  0.577003576  0.50429937  0.599095694  0.703855898  0.72586686  0.517588964  0.571348765  0.614579023  0.584794997  0.501195128  0.514503818  0.519336815  0.662838213  0.522070194  0.644790906  0.68294395  0.611700438  0.505709899  0.530349548  0.519968497  0.560814427  0.525581897  0.5282152  0.551792001  0.544913682  0.563900274  0.519316908  0.549992426  0.534933392  0.519490267  0.527268366  0.574766044  0.558936204  0.540557538  0.571707559  0.514165583  0.503316124  0.632655049  0.521972979  0.540128696  0.510823427  0.567478717  0.615382479  0.509078436  0.536442197  0.517245241  0.514042499  0.684057853  0.53608454  0.675844562  0.500288642  0.526608057  0.546395736  0.518243902  0.610507158  0.50591477  0.623292329  0.538067318  0.576815172  0.511278106  0.50147102  0.5162849  0.559816714  0.517210033  0.541678005  0.5383986  0.662250429  0.542230676  0.594859918  0.517566715  0.545969246  0.703013188  0.563895126  0.530619093  0.768892576  0.618950124  0.685979379  0.531106935  0.52213564  0.503862938  0.595412189  0.514975017  0.527872078  0.516243786  0.61073779  0.577618808  0.641574237  0.668272129  0.556935404  0.50429072  0.553380623  0.519817064  0.520502668  0.747925352  0.506323903  0.515521944  0.560967971  0.502019046  0.502168432  0.508405289  0.549942315  0.552680081  0.512091882  0.525090243  0.510310623  0.535092854  0.504961734  0.547831777  0.500594126  0.500525521  0.517306725  0.503515993  0.503393339  0.538647016  0.525323392  0.577095808  0.555933739  0.514269179  0.536907132  0.5010542  0.567757456  0.583958778  0.575045829 |
