# Supplementary material for: Systematic analyses of a novel lncRNA‐associated signature as the prognostic biomarker for Hepatocellular Carcinoma
Source: Cancer Med. 2018 May 15;7(7):3240–56. doi: 10.1002/cam4.1541 (PMC6051236; doi:10.1002/cam4.1541)
Supplement: Supplementary file 3 [file CAM4-7-3240-s003.doc]

**Supplementery Table 3: GO term and KEGG Pathway of co-expressed mRNAs in HCC**

| **Category** | **ID** | **Term** | **No. of Genes** | **enrichment** | **-lgP** | **-lgFDR** |
| --- | --- | --- | --- | --- | --- | --- |
| **GO** | GO:0044281 | small molecule metabolic process | 134 | 9.399 | 87.035 | 83.750 |
|  | GO:0034641 | cellular nitrogen compound metabolic process | 32 | 16.537 | 27.871 | 24.886 |
|  | GO:0006955 | immune response | 39 | 10.623 | 26.456 | 23.648 |
|  | GO:0044255 | cellular lipid metabolic process | 26 | 16.796 | 22.824 | 20.140 |
|  | GO:0006805 | xenobiotic metabolic process | 25 | 17.320 | 22.295 | 19.708 |
|  | GO:0045087 | innate immune response | 36 | 6.213 | 16.620 | 14.113 |
|  | GO:0050852 | T cell receptor signaling pathway | 17 | 18.899 | 15.850 | 13.410 |
|  | GO:0006635 | fatty acid beta-oxidation | 13 | 32.708 | 15.568 | 13.186 |
|  | GO:0007165 | signal transduction | 45 | 4.177 | 14.248 | 11.926 |
|  | GO:0008206 | bile acid metabolic process | 12 | 31.869 | 14.211 | 11.926 |
|  | GO:0007596 | blood coagulation | 28 | 5.757 | 12.113 | 9.869 |
|  | GO:0051607 | defense response to virus | 17 | 11.287 | 11.978 | 9.772 |
|  | GO:0031295 | T cell costimulation | 13 | 18.278 | 11.930 | 9.758 |
|  | GO:0006631 | fatty acid metabolic process | 12 | 20.487 | 11.644 | 9.505 |
|  | GO:0055085 | transmembrane transport | 29 | 5.154 | 11.348 | 9.239 |
|  | GO:0002504 | antigen processing and presentation of peptide or polysaccharide antigen via MHC class II | 7 | 66.925 | 11.208 | 9.127 |
|  | GO:0060333 | interferon-gamma-mediated signaling pathway | 12 | 17.651 | 10.823 | 8.768 |
|  | GO:0019373 | epoxygenase P450 pathway | 7 | 60.841 | 10.773 | 8.743 |
|  | GO:0009083 | branched-chain amino acid catabolic process | 8 | 42.492 | 10.660 | 8.665 |
|  | GO:0017144 | drug metabolic process | 9 | 31.869 | 10.649 | 8.665 |
|  | GO:0055114 | oxidation-reduction process | 13 | 11.951 | 9.488 | 7.525 |
|  | GO:0097267 | omega-hydroxylase P450 pathway | 6 | 63.738 | 9.379 | 7.436 |
|  | GO:0006629 | lipid metabolic process | 16 | 8.269 | 9.211 | 7.287 |
|  | GO:0006958 | complement activation, classical pathway | 9 | 20.487 | 8.731 | 6.844 |
|  | GO:0042110 | T cell activation | 9 | 20.487 | 8.731 | 6.844 |
|  | GO:0006508 | proteolysis | 24 | 4.702 | 8.597 | 6.727 |
|  | GO:0033539 | fatty acid beta-oxidation using acyl-CoA dehydrogenase | 5 | 79.673 | 8.533 | 6.710 |
|  | GO:0046487 | glyoxylate metabolic process | 5 | 79.673 | 8.533 | 6.710 |
|  | GO:0046951 | ketone body biosynthetic process | 5 | 79.673 | 8.533 | 6.710 |
|  | GO:0042493 | response to drug | 18 | 6.281 | 8.401 | 6.593 |
|  | GO:0019221 | cytokine-mediated signaling pathway | 16 | 7.082 | 8.221 | 6.427 |
|  | GO:0019882 | antigen processing and presentation | 8 | 22.496 | 8.106 | 6.336 |
|  | GO:0006699 | bile acid biosynthetic process | 7 | 30.420 | 8.103 | 6.336 |
|  | GO:0050776 | regulation of immune response | 11 | 11.817 | 7.979 | 6.225 |
|  | GO:0042157 | lipoprotein metabolic process | 8 | 20.672 | 7.790 | 6.049 |
|  | GO:0014070 | response to organic cyclic compound | 8 | 17.787 | 7.240 | 5.511 |
|  | GO:0042102 | positive regulation of T cell proliferation | 8 | 16.997 | 7.076 | 5.363 |
|  | GO:0042730 | fibrinolysis | 6 | 31.869 | 7.069 | 5.363 |
|  | GO:0042738 | exogenous drug catabolic process | 5 | 47.804 | 6.925 | 5.230 |
|  | GO:0030168 | platelet activation | 14 | 6.561 | 6.781 | 5.097 |
|  | GO:0019886 | antigen processing and presentation of exogenous peptide antigen via MHC class II | 10 | 10.392 | 6.710 | 5.037 |
|  | GO:0019369 | arachidonic acid metabolic process | 8 | 15.297 | 6.699 | 5.037 |
|  | GO:0006069 | ethanol oxidation | 5 | 43.458 | 6.665 | 5.013 |
|  | GO:0051289 | protein homotetramerization | 8 | 14.997 | 6.629 | 4.997 |
|  | GO:0046950 | cellular ketone body metabolic process | 4 | 76.486 | 6.629 | 4.997 |
|  | GO:0006917 | induction of apoptosis | 13 | 6.829 | 6.501 | 4.879 |
|  | GO:0006956 | complement activation | 7 | 18.590 | 6.469 | 4.855 |
|  | GO:0001916 | positive regulation of T cell mediated cytotoxicity | 5 | 39.836 | 6.435 | 4.840 |
|  | GO:0045060 | negative thymic T cell selection | 5 | 39.836 | 6.435 | 4.840 |
|  | GO:0006979 | response to oxidative stress | 10 | 9.466 | 6.321 | 4.734 |
|  | GO:0042632 | cholesterol homeostasis | 8 | 13.419 | 6.239 | 4.661 |
|  | GO:0006915 | apoptotic process | 24 | 3.509 | 6.180 | 4.610 |
|  | GO:0006006 | glucose metabolic process | 10 | 9.105 | 6.160 | 4.599 |
|  | GO:0006968 | cellular defense response | 8 | 12.748 | 6.061 | 4.508 |
|  | GO:0000096 | sulfur amino acid metabolic process | 6 | 22.063 | 6.006 | 4.461 |
|  | GO:0042594 | response to starvation | 6 | 21.246 | 5.901 | 4.364 |
|  | GO:0006094 | gluconeogenesis | 7 | 15.210 | 5.838 | 4.310 |
|  | GO:0030335 | positive regulation of cell migration | 10 | 8.387 | 5.824 | 4.310 |
|  | GO:0032496 | response to lipopolysaccharide | 10 | 8.387 | 5.824 | 4.310 |
|  | GO:0043030 | regulation of macrophage activation | 4 | 54.633 | 5.791 | 4.286 |
|  | GO:0010951 | negative regulation of endopeptidase activity | 8 | 11.767 | 5.786 | 4.286 |
|  | GO:0006935 | chemotaxis | 10 | 8.242 | 5.754 | 4.261 |
|  | GO:0006952 | defense response | 8 | 11.589 | 5.734 | 4.254 |
|  | GO:0051384 | response to glucocorticoid stimulus | 8 | 11.589 | 5.734 | 4.254 |
|  | GO:0007584 | response to nutrient | 8 | 11.416 | 5.682 | 4.210 |
|  | GO:0045944 | positive regulation of transcription from RNA polymerase II promoter | 24 | 3.241 | 5.565 | 4.100 |
|  | GO:0035335 | peptidyl-tyrosine dephosphorylation | 7 | 13.658 | 5.507 | 4.048 |
|  | GO:0008150 | biological_process | 22 | 3.403 | 5.450 | 3.997 |
|  | GO:0007166 | cell surface receptor signaling pathway | 11 | 6.699 | 5.421 | 3.974 |
|  | GO:0006579 | amino-acid betaine catabolic process | 3 | 95.607 | 5.341 | 3.919 |
|  | GO:0009436 | glyoxylate catabolic process | 3 | 95.607 | 5.341 | 3.919 |
|  | GO:0019448 | L-cysteine catabolic process | 3 | 95.607 | 5.341 | 3.919 |
|  | GO:0032787 | monocarboxylic acid metabolic process | 3 | 95.607 | 5.341 | 3.919 |
|  | GO:0070328 | triglyceride homeostasis | 5 | 25.160 | 5.294 | 3.878 |
|  | GO:0006853 | carnitine shuttle | 4 | 42.492 | 5.242 | 3.837 |
|  | GO:0045059 | positive thymic T cell selection | 4 | 42.492 | 5.242 | 3.837 |
|  | GO:0045471 | response to ethanol | 8 | 9.933 | 5.213 | 3.815 |
|  | GO:0019835 | cytolysis | 5 | 23.902 | 5.173 | 3.780 |
|  | GO:0008202 | steroid metabolic process | 7 | 11.951 | 5.103 | 3.716 |
|  | GO:0009791 | post-embryonic development | 8 | 9.561 | 5.086 | 3.704 |
|  | GO:0006869 | lipid transport | 7 | 11.741 | 5.050 | 3.674 |
|  | GO:0006470 | protein dephosphorylation | 9 | 7.822 | 4.988 | 3.616 |
|  | GO:0006954 | inflammatory response | 14 | 4.537 | 4.852 | 3.486 |
|  | GO:0033540 | fatty acid beta-oxidation using acyl-CoA oxidase | 4 | 34.766 | 4.831 | 3.482 |
|  | GO:0033559 | unsaturated fatty acid metabolic process | 4 | 34.766 | 4.831 | 3.482 |
|  | GO:0036109 | alpha-linolenic acid metabolic process | 4 | 34.766 | 4.831 | 3.482 |
|  | GO:0009615 | response to virus | 9 | 7.482 | 4.827 | 3.482 |
|  | GO:0048661 | positive regulation of smooth muscle cell proliferation | 6 | 13.991 | 4.774 | 3.433 |
|  | GO:0051919 | positive regulation of fibrinolysis | 3 | 71.705 | 4.743 | 3.407 |
|  | GO:0019048 | virus-host interaction | 14 | 4.332 | 4.621 | 3.290 |
|  | GO:0008203 | cholesterol metabolic process | 7 | 10.140 | 4.616 | 3.290 |
|  | GO:0007259 | JAK-STAT cascade | 5 | 18.386 | 4.568 | 3.251 |
|  | GO:0030217 | T cell differentiation | 5 | 18.386 | 4.568 | 3.251 |
|  | GO:0007155 | cell adhesion | 17 | 3.580 | 4.499 | 3.187 |
|  | GO:0043065 | positive regulation of apoptotic process | 11 | 5.338 | 4.467 | 3.160 |
|  | GO:0001889 | liver development | 7 | 9.426 | 4.404 | 3.103 |
|  | GO:0050690 | regulation of defense response to virus by virus | 5 | 17.073 | 4.401 | 3.103 |
|  | GO:0000038 | very long-chain fatty acid metabolic process | 4 | 27.316 | 4.360 | 3.076 |
|  | GO:0016311 | dephosphorylation | 4 | 27.316 | 4.360 | 3.076 |
|  | GO:0030101 | natural killer cell activation | 4 | 27.316 | 4.360 | 3.076 |
|  | GO:0007267 | cell-cell signaling | 12 | 4.741 | 4.350 | 3.076 |
|  | GO:0006552 | leucine catabolic process | 3 | 57.364 | 4.348 | 3.076 |
|  | GO:0019626 | short-chain fatty acid catabolic process | 3 | 57.364 | 4.348 | 3.076 |
|  | GO:0006633 | fatty acid biosynthetic process | 6 | 11.707 | 4.312 | 3.043 |
|  | GO:0006898 | receptor-mediated endocytosis | 6 | 11.473 | 4.260 | 2.996 |
|  | GO:0008209 | androgen metabolic process | 4 | 25.495 | 4.229 | 2.973 |
|  | GO:0051881 | regulation of mitochondrial membrane potential | 4 | 25.495 | 4.229 | 2.973 |
|  | GO:0007264 | small GTPase mediated signal transduction | 14 | 3.984 | 4.213 | 2.961 |
|  | GO:0055086 | nucleobase-containing small molecule metabolic process | 7 | 8.692 | 4.170 | 2.922 |
|  | GO:0034097 | response to cytokine stimulus | 6 | 11.032 | 4.160 | 2.916 |
|  | GO:0032480 | negative regulation of type I interferon production | 5 | 14.939 | 4.105 | 2.865 |
|  | GO:0045893 | positive regulation of transcription, DNA-dependent | 17 | 3.310 | 4.064 | 2.837 |
|  | GO:0001561 | fatty acid alpha-oxidation | 3 | 47.804 | 4.051 | 2.837 |
|  | GO:0006066 | alcohol metabolic process | 3 | 47.804 | 4.051 | 2.837 |
|  | GO:0009086 | methionine biosynthetic process | 3 | 47.804 | 4.051 | 2.837 |
|  | GO:0015833 | peptide transport | 3 | 47.804 | 4.051 | 2.837 |
|  | GO:0016098 | monoterpenoid metabolic process | 3 | 47.804 | 4.051 | 2.837 |
|  | GO:0090181 | regulation of cholesterol metabolic process | 3 | 47.804 | 4.051 | 2.837 |
|  | GO:0001676 | long-chain fatty acid metabolic process | 4 | 22.496 | 3.994 | 2.788 |
|  | GO:0015721 | bile acid and bile salt transport | 4 | 22.496 | 3.994 | 2.788 |
|  | GO:0006461 | protein complex assembly | 8 | 6.769 | 3.970 | 2.768 |
|  | GO:0008285 | negative regulation of cell proliferation | 14 | 3.739 | 3.910 | 2.713 |
|  | GO:0006641 | triglyceride metabolic process | 5 | 13.658 | 3.909 | 2.713 |
|  | GO:0005978 | glycogen biosynthetic process | 4 | 21.246 | 3.889 | 2.701 |
|  | GO:0007597 | blood coagulation, intrinsic pathway | 4 | 21.246 | 3.889 | 2.701 |
|  | GO:0043687 | post-translational protein modification | 10 | 5.085 | 3.880 | 2.695 |
|  | GO:0032088 | negative regulation of NF-kappaB transcription factor activity | 6 | 9.723 | 3.842 | 2.660 |
|  | GO:0019441 | tryptophan catabolic process to kynurenine | 3 | 40.974 | 3.811 | 2.640 |
|  | GO:0045588 | positive regulation of gamma-delta T cell differentiation | 3 | 40.974 | 3.811 | 2.640 |
|  | GO:0048304 | positive regulation of isotype switching to IgG isotypes | 3 | 40.974 | 3.811 | 2.640 |
|  | GO:0032355 | response to estradiol stimulus | 6 | 9.404 | 3.759 | 2.591 |
|  | GO:0051591 | response to cAMP | 5 | 12.580 | 3.731 | 2.566 |
|  | GO:0050850 | positive regulation of calcium-mediated signaling | 4 | 19.121 | 3.697 | 2.535 |
|  | GO:0042542 | response to hydrogen peroxide | 5 | 12.257 | 3.675 | 2.517 |
|  | GO:0005975 | carbohydrate metabolic process | 13 | 3.755 | 3.650 | 2.495 |
|  | GO:0051056 | regulation of small GTPase mediated signal transduction | 9 | 5.279 | 3.614 | 2.472 |
|  | GO:0000098 | sulfur amino acid catabolic process | 3 | 35.853 | 3.610 | 2.472 |
|  | GO:0010269 | response to selenium ion | 3 | 35.853 | 3.610 | 2.472 |
|  | GO:0032609 | interferon-gamma production | 3 | 35.853 | 3.610 | 2.472 |
|  | GO:0060396 | growth hormone receptor signaling pathway | 3 | 35.853 | 3.610 | 2.472 |
|  | GO:0071346 | cellular response to interferon-gamma | 4 | 18.211 | 3.608 | 2.472 |
|  | GO:0043066 | negative regulation of apoptotic process | 16 | 3.161 | 3.589 | 2.456 |
|  | GO:0007610 | behavior | 5 | 11.659 | 3.568 | 2.438 |
|  | GO:0050731 | positive regulation of peptidyl-tyrosine phosphorylation | 6 | 8.562 | 3.527 | 2.400 |
|  | GO:0002474 | antigen processing and presentation of peptide antigen via MHC class I | 7 | 6.899 | 3.520 | 2.396 |
|  | GO:0009636 | response to toxic substance | 6 | 8.314 | 3.455 | 2.334 |
|  | GO:0006559 | L-phenylalanine catabolic process | 3 | 31.869 | 3.438 | 2.323 |
|  | GO:0070989 | oxidative demethylation | 3 | 31.869 | 3.438 | 2.323 |
|  | GO:0008015 | blood circulation | 5 | 10.864 | 3.418 | 2.306 |
|  | GO:0071356 | cellular response to tumor necrosis factor | 5 | 10.623 | 3.370 | 2.286 |
|  | GO:0002302 | CD8-positive, alpha-beta T cell differentiation involved in immune response | 2 | 95.607 | 3.360 | 2.286 |
|  | GO:0002481 | antigen processing and presentation of exogenous protein antigen via MHC class Ib, TAP-dependent | 2 | 95.607 | 3.360 | 2.286 |
|  | GO:0006116 | NADH oxidation | 2 | 95.607 | 3.360 | 2.286 |
|  | GO:0009450 | gamma-aminobutyric acid catabolic process | 2 | 95.607 | 3.360 | 2.286 |
|  | GO:0019265 | glycine biosynthetic process, by transamination of glyoxylate | 2 | 95.607 | 3.360 | 2.286 |
|  | GO:0032305 | positive regulation of icosanoid secretion | 2 | 95.607 | 3.360 | 2.286 |
|  | GO:0042412 | taurine biosynthetic process | 2 | 95.607 | 3.360 | 2.286 |
|  | GO:0043551 | regulation of phosphatidylinositol 3-kinase activity | 2 | 95.607 | 3.360 | 2.286 |
|  | GO:0046395 | carboxylic acid catabolic process | 2 | 95.607 | 3.360 | 2.286 |
|  | GO:0046967 | cytosol to ER transport | 2 | 95.607 | 3.360 | 2.286 |
|  | GO:0051180 | vitamin transport | 2 | 95.607 | 3.360 | 2.286 |
|  | GO:0055090 | acylglycerol homeostasis | 2 | 95.607 | 3.360 | 2.286 |
|  | GO:0072308 | negative regulation of metanephric nephron tubule epithelial cell differentiation | 2 | 95.607 | 3.360 | 2.286 |
|  | GO:0045597 | positive regulation of cell differentiation | 4 | 15.297 | 3.298 | 2.227 |
|  | GO:0051918 | negative regulation of fibrinolysis | 3 | 28.682 | 3.286 | 2.218 |
|  | GO:0007186 | G-protein coupled receptor signaling pathway | 12 | 3.665 | 3.271 | 2.206 |
|  | GO:0002479 | antigen processing and presentation of exogenous peptide antigen via MHC class I, TAP-dependent | 6 | 7.649 | 3.253 | 2.192 |
|  | GO:0050900 | leukocyte migration | 7 | 6.255 | 3.252 | 2.192 |
|  | GO:0006665 | sphingolipid metabolic process | 6 | 7.548 | 3.221 | 2.164 |
|  | GO:0006569 | tryptophan catabolic process | 3 | 26.075 | 3.151 | 2.115 |
|  | GO:0006702 | androgen biosynthetic process | 3 | 26.075 | 3.151 | 2.115 |
|  | GO:0016064 | immunoglobulin mediated immune response | 3 | 26.075 | 3.151 | 2.115 |
|  | GO:0017187 | peptidyl-glutamic acid carboxylation | 3 | 26.075 | 3.151 | 2.115 |
|  | GO:0046135 | pyrimidine nucleoside catabolic process | 3 | 26.075 | 3.151 | 2.115 |
|  | GO:0046641 | positive regulation of alpha-beta T cell proliferation | 3 | 26.075 | 3.151 | 2.115 |
|  | GO:0050868 | negative regulation of T cell activation | 3 | 26.075 | 3.151 | 2.115 |
|  | GO:0060068 | vagina development | 3 | 26.075 | 3.151 | 2.115 |
|  | GO:0032868 | response to insulin stimulus | 5 | 9.561 | 3.150 | 2.115 |
|  | GO:0042590 | antigen processing and presentation of exogenous peptide antigen via MHC class I | 6 | 7.261 | 3.128 | 2.096 |
|  | GO:0010332 | response to gamma radiation | 4 | 13.658 | 3.099 | 2.069 |
|  | GO:0002576 | platelet degranulation | 6 | 7.082 | 3.069 | 2.041 |
|  | GO:0042981 | regulation of apoptotic process | 9 | 4.435 | 3.041 | 2.016 |
|  | GO:0030522 | intracellular receptor signaling pathway | 4 | 13.187 | 3.039 | 2.016 |
|  | GO:0042574 | retinal metabolic process | 3 | 23.902 | 3.030 | 2.009 |
|  | GO:0006644 | phospholipid metabolic process | 8 | 4.935 | 3.008 | 1.990 |
|  | GO:0045429 | positive regulation of nitric oxide biosynthetic process | 4 | 12.748 | 2.980 | 1.964 |
|  | GO:0007586 | digestion | 5 | 8.692 | 2.953 | 1.940 |
|  | GO:0032729 | positive regulation of interferon-gamma production | 4 | 12.336 | 2.924 | 1.917 |
|  | GO:0050853 | B cell receptor signaling pathway | 4 | 12.336 | 2.924 | 1.917 |
|  | GO:0006809 | nitric oxide biosynthetic process | 3 | 22.063 | 2.919 | 1.917 |
|  | GO:0030889 | negative regulation of B cell proliferation | 3 | 22.063 | 2.919 | 1.917 |
|  | GO:0032259 | methylation | 3 | 22.063 | 2.919 | 1.917 |
|  | GO:0001523 | retinoid metabolic process | 5 | 8.536 | 2.916 | 1.917 |
|  | GO:0001766 | membrane raft polarization | 2 | 63.738 | 2.886 | 1.914 |
|  | GO:0003095 | pressure natriuresis | 2 | 63.738 | 2.886 | 1.914 |
|  | GO:0003340 | negative regulation of mesenchymal to epithelial transition involved in metanephros morphogenesis | 2 | 63.738 | 2.886 | 1.914 |
|  | GO:0010044 | response to aluminum ion | 2 | 63.738 | 2.886 | 1.914 |
|  | GO:0010248 | establishment or maintenance of transmembrane electrochemical gradient | 2 | 63.738 | 2.886 | 1.914 |
|  | GO:0014904 | myotube cell development | 2 | 63.738 | 2.886 | 1.914 |
|  | GO:0019254 | carnitine metabolic process, CoA-linked | 2 | 63.738 | 2.886 | 1.914 |
|  | GO:0030573 | bile acid catabolic process | 2 | 63.738 | 2.886 | 1.914 |
|  | GO:0036101 | leukotriene B4 catabolic process | 2 | 63.738 | 2.886 | 1.914 |
|  | GO:0045590 | negative regulation of regulatory T cell differentiation | 2 | 63.738 | 2.886 | 1.914 |
|  | GO:0046477 | glycosylceramide catabolic process | 2 | 63.738 | 2.886 | 1.914 |
|  | GO:0050855 | regulation of B cell receptor signaling pathway | 2 | 63.738 | 2.886 | 1.914 |
|  | GO:0070233 | negative regulation of T cell apoptotic process | 2 | 63.738 | 2.886 | 1.914 |
|  | GO:0000122 | negative regulation of transcription from RNA polymerase II promoter | 15 | 2.817 | 2.832 | 1.863 |
|  | GO:0006637 | acyl-CoA metabolic process | 3 | 20.487 | 2.818 | 1.861 |
|  | GO:0019395 | fatty acid oxidation | 3 | 20.487 | 2.818 | 1.861 |
|  | GO:0021772 | olfactory bulb development | 3 | 20.487 | 2.818 | 1.861 |
|  | GO:0030301 | cholesterol transport | 3 | 20.487 | 2.818 | 1.861 |
|  | GO:0042573 | retinoic acid metabolic process | 3 | 20.487 | 2.818 | 1.861 |
|  | GO:0048009 | insulin-like growth factor receptor signaling pathway | 3 | 20.487 | 2.818 | 1.861 |
|  | GO:0044267 | cellular protein metabolic process | 15 | 2.785 | 2.780 | 1.825 |
|  | GO:0007050 | cell cycle arrest | 7 | 5.229 | 2.774 | 1.821 |
|  | GO:0001933 | negative regulation of protein phosphorylation | 4 | 11.248 | 2.766 | 1.815 |
|  | GO:0030890 | positive regulation of B cell proliferation | 4 | 10.927 | 2.717 | 1.768 |
|  | GO:0000278 | mitotic cell cycle | 12 | 3.161 | 2.688 | 1.742 |
|  | GO:0016485 | protein processing | 4 | 10.623 | 2.669 | 1.724 |
|  | GO:0007169 | transmembrane receptor protein tyrosine kinase signaling pathway | 5 | 7.469 | 2.646 | 1.705 |
|  | GO:0006928 | cellular component movement | 6 | 5.914 | 2.646 | 1.705 |
|  | GO:0006778 | porphyrin-containing compound metabolic process | 3 | 17.926 | 2.637 | 1.702 |
|  | GO:0042523 | positive regulation of tyrosine phosphorylation of Stat5 protein | 3 | 17.926 | 2.637 | 1.702 |
|  | GO:0044130 | negative regulation of growth of symbiont in host | 3 | 17.926 | 2.637 | 1.702 |
|  | GO:0006953 | acute-phase response | 4 | 10.336 | 2.623 | 1.690 |
|  | GO:0006879 | cellular iron ion homeostasis | 5 | 7.354 | 2.615 | 1.688 |
|  | GO:0060337 | type I interferon-mediated signaling pathway | 5 | 7.354 | 2.615 | 1.688 |
|  | GO:0002669 | positive regulation of T cell anergy | 2 | 47.804 | 2.588 | 1.688 |
|  | GO:0002819 | regulation of adaptive immune response | 2 | 47.804 | 2.588 | 1.688 |
|  | GO:0006084 | acetyl-CoA metabolic process | 2 | 47.804 | 2.588 | 1.688 |
|  | GO:0016139 | glycoside catabolic process | 2 | 47.804 | 2.588 | 1.688 |
|  | GO:0019439 | aromatic compound catabolic process | 2 | 47.804 | 2.588 | 1.688 |
|  | GO:0019556 | histidine catabolic process to glutamate and formamide | 2 | 47.804 | 2.588 | 1.688 |
|  | GO:0019557 | histidine catabolic process to glutamate and formate | 2 | 47.804 | 2.588 | 1.688 |
|  | GO:0031998 | regulation of fatty acid beta-oxidation | 2 | 47.804 | 2.588 | 1.688 |
|  | GO:0035519 | protein K29-linked ubiquitination | 2 | 47.804 | 2.588 | 1.688 |
|  | GO:0043372 | positive regulation of CD4-positive, alpha-beta T cell differentiation | 2 | 47.804 | 2.588 | 1.688 |
|  | GO:0045345 | positive regulation of MHC class I biosynthetic process | 2 | 47.804 | 2.588 | 1.688 |
|  | GO:0046642 | negative regulation of alpha-beta T cell proliferation | 2 | 47.804 | 2.588 | 1.688 |
|  | GO:0046952 | ketone body catabolic process | 2 | 47.804 | 2.588 | 1.688 |
|  | GO:0060335 | positive regulation of interferon-gamma-mediated signaling pathway | 2 | 47.804 | 2.588 | 1.688 |
|  | GO:0070189 | kynurenine metabolic process | 2 | 47.804 | 2.588 | 1.688 |
|  | GO:0071800 | podosome assembly | 2 | 47.804 | 2.588 | 1.688 |
|  | GO:0002250 | adaptive immune response | 3 | 16.872 | 2.556 | 1.664 |
|  | GO:0019915 | lipid storage | 3 | 16.872 | 2.556 | 1.664 |
|  | GO:0046686 | response to cadmium ion | 3 | 16.872 | 2.556 | 1.664 |
|  | GO:0060612 | adipose tissue development | 3 | 16.872 | 2.556 | 1.664 |
|  | GO:0000080 | G1 phase of mitotic cell cycle | 4 | 9.806 | 2.535 | 1.648 |
|  | GO:0009725 | response to hormone stimulus | 4 | 9.806 | 2.535 | 1.648 |
|  | GO:0031018 | endocrine pancreas development | 4 | 9.806 | 2.535 | 1.648 |
|  | GO:0071222 | cellular response to lipopolysaccharide | 5 | 6.928 | 2.496 | 1.610 |
|  | GO:0043434 | response to peptide hormone stimulus | 4 | 9.561 | 2.493 | 1.609 |
|  | GO:0006691 | leukotriene metabolic process | 3 | 15.935 | 2.481 | 1.600 |
|  | GO:0051603 | proteolysis involved in cellular protein catabolic process | 3 | 15.935 | 2.481 | 1.600 |
|  | GO:0006959 | humoral immune response | 4 | 9.328 | 2.452 | 1.575 |
|  | GO:0019432 | triglyceride biosynthetic process | 4 | 9.328 | 2.452 | 1.575 |
|  | GO:0019722 | calcium-mediated signaling | 4 | 9.105 | 2.412 | 1.541 |
|  | GO:0006783 | heme biosynthetic process | 3 | 15.096 | 2.409 | 1.541 |
|  | GO:0009306 | protein secretion | 3 | 15.096 | 2.409 | 1.541 |
|  | GO:0019433 | triglyceride catabolic process | 3 | 15.096 | 2.409 | 1.541 |
|  | GO:0042572 | retinol metabolic process | 3 | 15.096 | 2.409 | 1.541 |
|  | GO:0002830 | positive regulation of type 2 immune response | 2 | 38.243 | 2.369 | 1.527 |
|  | GO:0006548 | histidine catabolic process | 2 | 38.243 | 2.369 | 1.527 |
|  | GO:0006573 | valine metabolic process | 2 | 38.243 | 2.369 | 1.527 |
|  | GO:0006642 | triglyceride mobilization | 2 | 38.243 | 2.369 | 1.527 |
|  | GO:0015872 | dopamine transport | 2 | 38.243 | 2.369 | 1.527 |
|  | GO:0019060 | intracellular transport of viral proteins in host cell | 2 | 38.243 | 2.369 | 1.527 |
|  | GO:0020027 | hemoglobin metabolic process | 2 | 38.243 | 2.369 | 1.527 |
|  | GO:0032747 | positive regulation of interleukin-23 production | 2 | 38.243 | 2.369 | 1.527 |
|  | GO:0033622 | integrin activation | 2 | 38.243 | 2.369 | 1.527 |
|  | GO:0042158 | lipoprotein biosynthetic process | 2 | 38.243 | 2.369 | 1.527 |
|  | GO:0042737 | drug catabolic process | 2 | 38.243 | 2.369 | 1.527 |
|  | GO:0042866 | pyruvate biosynthetic process | 2 | 38.243 | 2.369 | 1.527 |
|  | GO:0044539 | long-chain fatty acid import | 2 | 38.243 | 2.369 | 1.527 |
|  | GO:0045329 | carnitine biosynthetic process | 2 | 38.243 | 2.369 | 1.527 |
|  | GO:0045580 | regulation of T cell differentiation | 2 | 38.243 | 2.369 | 1.527 |
|  | GO:0050863 | regulation of T cell activation | 2 | 38.243 | 2.369 | 1.527 |
|  | GO:0050996 | positive regulation of lipid catabolic process | 2 | 38.243 | 2.369 | 1.527 |
|  | GO:0007031 | peroxisome organization | 3 | 14.341 | 2.342 | 1.506 |
|  | GO:0042326 | negative regulation of phosphorylation | 3 | 14.341 | 2.342 | 1.506 |
|  | GO:0090398 | cellular senescence | 3 | 14.341 | 2.342 | 1.506 |
|  | GO:0006260 | DNA replication | 7 | 4.403 | 2.333 | 1.498 |
|  | GO:0050680 | negative regulation of epithelial cell proliferation | 4 | 8.498 | 2.298 | 1.465 |
|  | GO:0006919 | activation of cysteine-type endopeptidase activity involved in apoptotic process | 5 | 6.129 | 2.256 | 1.424 |
|  | GO:0006464 | cellular protein modification process | 6 | 4.945 | 2.241 | 1.410 |
|  | GO:0022617 | extracellular matrix disassembly | 5 | 6.051 | 2.231 | 1.402 |
|  | GO:0008284 | positive regulation of cell proliferation | 12 | 2.791 | 2.226 | 1.398 |
|  | GO:0001867 | complement activation, lectin pathway | 2 | 31.869 | 2.196 | 1.395 |
|  | GO:0002456 | T cell mediated immunity | 2 | 31.869 | 2.196 | 1.395 |
|  | GO:0002639 | positive regulation of immunoglobulin production | 2 | 31.869 | 2.196 | 1.395 |
|  | GO:0003091 | renal water homeostasis | 2 | 31.869 | 2.196 | 1.395 |
|  | GO:0006475 | internal protein amino acid acetylation | 2 | 31.869 | 2.196 | 1.395 |
|  | GO:0006544 | glycine metabolic process | 2 | 31.869 | 2.196 | 1.395 |
|  | GO:0006729 | tetrahydrobiopterin biosynthetic process | 2 | 31.869 | 2.196 | 1.395 |
|  | GO:0010898 | positive regulation of triglyceride catabolic process | 2 | 31.869 | 2.196 | 1.395 |
|  | GO:0019885 | antigen processing and presentation of endogenous peptide antigen via MHC class I | 2 | 31.869 | 2.196 | 1.395 |
|  | GO:0031639 | plasminogen activation | 2 | 31.869 | 2.196 | 1.395 |
|  | GO:0032873 | negative regulation of stress-activated MAPK cascade | 2 | 31.869 | 2.196 | 1.395 |
|  | GO:0033261 | regulation of S phase | 2 | 31.869 | 2.196 | 1.395 |
|  | GO:0034393 | positive regulation of smooth muscle cell apoptotic process | 2 | 31.869 | 2.196 | 1.395 |
|  | GO:0046483 | heterocycle metabolic process | 2 | 31.869 | 2.196 | 1.395 |
|  | GO:0050667 | homocysteine metabolic process | 2 | 31.869 | 2.196 | 1.395 |
|  | GO:0051347 | positive regulation of transferase activity | 2 | 31.869 | 2.196 | 1.395 |
|  | GO:0060340 | positive regulation of type I interferon-mediated signaling pathway | 2 | 31.869 | 2.196 | 1.395 |
|  | GO:0071354 | cellular response to interleukin-6 | 2 | 31.869 | 2.196 | 1.395 |
|  | GO:0006206 | pyrimidine nucleobase metabolic process | 3 | 12.470 | 2.161 | 1.363 |
|  | GO:0070098 | chemokine-mediated signaling pathway | 3 | 12.470 | 2.161 | 1.363 |
|  | GO:0045766 | positive regulation of angiogenesis | 5 | 5.691 | 2.113 | 1.316 |
|  | GO:0050918 | positive chemotaxis | 3 | 11.951 | 2.106 | 1.312 |
|  | GO:0060397 | JAK-STAT cascade involved in growth hormone signaling pathway | 3 | 11.951 | 2.106 | 1.312 |
|  | GO:0007269 | neurotransmitter secretion | 4 | 7.354 | 2.064 | 1.286 |
|  | GO:0000188 | inactivation of MAPK activity | 3 | 11.473 | 2.054 | 1.286 |
|  | GO:0001937 | negative regulation of endothelial cell proliferation | 3 | 11.473 | 2.054 | 1.286 |
|  | GO:0005977 | glycogen metabolic process | 3 | 11.473 | 2.054 | 1.286 |
|  | GO:0032870 | cellular response to hormone stimulus | 3 | 11.473 | 2.054 | 1.286 |
|  | GO:0070534 | protein K63-linked ubiquitination | 3 | 11.473 | 2.054 | 1.286 |
|  | GO:0002925 | positive regulation of humoral immune response mediated by circulating immunoglobulin | 2 | 27.316 | 2.053 | 1.286 |
|  | GO:0006268 | DNA unwinding involved in replication | 2 | 27.316 | 2.053 | 1.286 |
|  | GO:0006690 | icosanoid metabolic process | 2 | 27.316 | 2.053 | 1.286 |
|  | GO:0009072 | aromatic amino acid family metabolic process | 2 | 27.316 | 2.053 | 1.286 |
|  | GO:0033762 | response to glucagon stimulus | 2 | 27.316 | 2.053 | 1.286 |
|  | GO:0035999 | tetrahydrofolate interconversion | 2 | 27.316 | 2.053 | 1.286 |
|  | GO:0042135 | neurotransmitter catabolic process | 2 | 27.316 | 2.053 | 1.286 |
|  | GO:0045084 | positive regulation of interleukin-12 biosynthetic process | 2 | 27.316 | 2.053 | 1.286 |
|  | GO:0045348 | positive regulation of MHC class II biosynthetic process | 2 | 27.316 | 2.053 | 1.286 |
|  | GO:0046689 | response to mercury ion | 2 | 27.316 | 2.053 | 1.286 |
|  | GO:0050862 | positive regulation of T cell receptor signaling pathway | 2 | 27.316 | 2.053 | 1.286 |
|  | GO:0055078 | sodium ion homeostasis | 2 | 27.316 | 2.053 | 1.286 |
|  | GO:0060351 | cartilage development involved in endochondral bone morphogenesis | 2 | 27.316 | 2.053 | 1.286 |
|  | GO:0060527 | prostate epithelial cord arborization involved in prostate glandular acinus morphogenesis | 2 | 27.316 | 2.053 | 1.286 |
|  | GO:0016525 | negative regulation of angiogenesis | 4 | 7.216 | 2.034 | 1.268 |
|  | GO:0048015 | phosphatidylinositol-mediated signaling | 6 | 4.447 | 2.007 | 1.243 |
|  | GO:0043200 | response to amino acid stimulus | 3 | 11.032 | 2.004 | 1.243 |
|  | GO:0051262 | protein tetramerization | 3 | 11.032 | 2.004 | 1.243 |
|  | GO:0007049 | cell cycle | 8 | 3.415 | 1.974 | 1.215 |
|  | GO:0010628 | positive regulation of gene expression | 6 | 4.379 | 1.974 | 1.215 |
|  | GO:0010033 | response to organic substance | 3 | 10.623 | 1.957 | 1.200 |
|  | GO:0031663 | lipopolysaccharide-mediated signaling pathway | 3 | 10.623 | 1.957 | 1.200 |
|  | GO:0018279 | protein N-linked glycosylation via asparagine | 5 | 5.196 | 1.940 | 1.191 |
|  | GO:0002262 | myeloid cell homeostasis | 2 | 23.902 | 1.931 | 1.191 |
|  | GO:0006105 | succinate metabolic process | 2 | 23.902 | 1.931 | 1.191 |
|  | GO:0006111 | regulation of gluconeogenesis | 2 | 23.902 | 1.931 | 1.191 |
|  | GO:0006546 | glycine catabolic process | 2 | 23.902 | 1.931 | 1.191 |
|  | GO:0030595 | leukocyte chemotaxis | 2 | 23.902 | 1.931 | 1.191 |
|  | GO:0032700 | negative regulation of interleukin-17 production | 2 | 23.902 | 1.931 | 1.191 |
|  | GO:0033673 | negative regulation of kinase activity | 2 | 23.902 | 1.931 | 1.191 |
|  | GO:0034383 | low-density lipoprotein particle clearance | 2 | 23.902 | 1.931 | 1.191 |
|  | GO:0035458 | cellular response to interferon-beta | 2 | 23.902 | 1.931 | 1.191 |
|  | GO:0045582 | positive regulation of T cell differentiation | 2 | 23.902 | 1.931 | 1.191 |
|  | GO:0070206 | protein trimerization | 2 | 23.902 | 1.931 | 1.191 |
|  | GO:0071391 | cellular response to estrogen stimulus | 2 | 23.902 | 1.931 | 1.191 |
|  | GO:0007603 | phototransduction, visible light | 4 | 6.709 | 1.918 | 1.180 |
|  | GO:0071260 | cellular response to mechanical stimulus | 4 | 6.709 | 1.918 | 1.180 |
|  | GO:0048706 | embryonic skeletal system development | 3 | 10.244 | 1.911 | 1.174 |
|  | GO:0000075 | cell cycle checkpoint | 6 | 4.249 | 1.909 | 1.174 |
|  | GO:0007568 | aging | 5 | 5.085 | 1.900 | 1.166 |
|  | GO:0009749 | response to glucose stimulus | 4 | 6.594 | 1.890 | 1.158 |
|  | GO:0006468 | protein phosphorylation | 10 | 2.829 | 1.885 | 1.154 |
|  | GO:0000187 | activation of MAPK activity | 5 | 5.032 | 1.880 | 1.150 |
|  | GO:0006099 | tricarboxylic acid cycle | 3 | 9.890 | 1.867 | 1.138 |
|  | GO:0006469 | negative regulation of protein kinase activity | 4 | 6.482 | 1.863 | 1.136 |
|  | GO:0008217 | regulation of blood pressure | 4 | 6.374 | 1.837 | 1.118 |
|  | GO:0002480 | antigen processing and presentation of exogenous peptide antigen via MHC class I, TAP-independent | 2 | 21.246 | 1.824 | 1.118 |
|  | GO:0006000 | fructose metabolic process | 2 | 21.246 | 1.824 | 1.118 |
|  | GO:0006107 | oxaloacetate metabolic process | 2 | 21.246 | 1.824 | 1.118 |
|  | GO:0006625 | protein targeting to peroxisome | 2 | 21.246 | 1.824 | 1.118 |
|  | GO:0006782 | protoporphyrinogen IX biosynthetic process | 2 | 21.246 | 1.824 | 1.118 |
|  | GO:0009605 | response to external stimulus | 2 | 21.246 | 1.824 | 1.118 |
|  | GO:0010225 | response to UV-C | 2 | 21.246 | 1.824 | 1.118 |
|  | GO:0015914 | phospholipid transport | 2 | 21.246 | 1.824 | 1.118 |
|  | GO:0031953 | negative regulation of protein autophosphorylation | 2 | 21.246 | 1.824 | 1.118 |
|  | GO:0042511 | positive regulation of tyrosine phosphorylation of Stat1 protein | 2 | 21.246 | 1.824 | 1.118 |
|  | GO:0045080 | positive regulation of chemokine biosynthetic process | 2 | 21.246 | 1.824 | 1.118 |
|  | GO:0048245 | eosinophil chemotaxis | 2 | 21.246 | 1.824 | 1.118 |
|  | GO:0050732 | negative regulation of peptidyl-tyrosine phosphorylation | 2 | 21.246 | 1.824 | 1.118 |
|  | GO:0051006 | positive regulation of lipoprotein lipase activity | 2 | 21.246 | 1.824 | 1.118 |
|  | GO:0060065 | uterus development | 2 | 21.246 | 1.824 | 1.118 |
|  | GO:2000045 | regulation of G1/S transition of mitotic cell cycle | 2 | 21.246 | 1.824 | 1.118 |
|  | GO:2000377 | regulation of reactive oxygen species metabolic process | 2 | 21.246 | 1.824 | 1.118 |
|  | GO:0006112 | energy reserve metabolic process | 5 | 4.878 | 1.822 | 1.117 |
|  | GO:0006271 | DNA strand elongation involved in DNA replication | 3 | 9.252 | 1.783 | 1.081 |
|  | GO:0009409 | response to cold | 3 | 9.252 | 1.783 | 1.081 |
|  | GO:0045071 | negative regulation of viral genome replication | 3 | 9.252 | 1.783 | 1.081 |
|  | GO:0006810 | transport | 10 | 2.732 | 1.779 | 1.078 |
|  | GO:0007005 | mitochondrion organization | 3 | 8.963 | 1.744 | 1.046 |
|  | GO:0032760 | positive regulation of tumor necrosis factor production | 3 | 8.963 | 1.744 | 1.046 |
|  | GO:0040007 | growth | 3 | 8.963 | 1.744 | 1.046 |
|  | GO:0006600 | creatine metabolic process | 2 | 19.121 | 1.731 | 1.043 |
|  | GO:0006855 | drug transmembrane transport | 2 | 19.121 | 1.731 | 1.043 |
|  | GO:0006882 | cellular zinc ion homeostasis | 2 | 19.121 | 1.731 | 1.043 |
|  | GO:0009100 | glycoprotein metabolic process | 2 | 19.121 | 1.731 | 1.043 |
|  | GO:0010613 | positive regulation of cardiac muscle hypertrophy | 2 | 19.121 | 1.731 | 1.043 |
|  | GO:0015695 | organic cation transport | 2 | 19.121 | 1.731 | 1.043 |
|  | GO:0019953 | sexual reproduction | 2 | 19.121 | 1.731 | 1.043 |
|  | GO:0043623 | cellular protein complex assembly | 2 | 19.121 | 1.731 | 1.043 |
|  | GO:0045579 | positive regulation of B cell differentiation | 2 | 19.121 | 1.731 | 1.043 |
|  | GO:0030317 | sperm motility | 3 | 8.692 | 1.706 | 1.019 |
|  | GO:0010629 | negative regulation of gene expression | 4 | 5.794 | 1.689 | 1.003 |
|  | GO:0009058 | biosynthetic process | 3 | 8.436 | 1.669 | 0.986 |
|  | GO:0030148 | sphingolipid biosynthetic process | 3 | 8.436 | 1.669 | 0.986 |
|  | GO:0001678 | cellular glucose homeostasis | 2 | 17.383 | 1.646 | 0.973 |
|  | GO:0001953 | negative regulation of cell-matrix adhesion | 2 | 17.383 | 1.646 | 0.973 |
|  | GO:0006195 | purine nucleotide catabolic process | 2 | 17.383 | 1.646 | 0.973 |
|  | GO:0006536 | glutamate metabolic process | 2 | 17.383 | 1.646 | 0.973 |
|  | GO:0006707 | cholesterol catabolic process | 2 | 17.383 | 1.646 | 0.973 |
|  | GO:0010039 | response to iron ion | 2 | 17.383 | 1.646 | 0.973 |
|  | GO:0014911 | positive regulation of smooth muscle cell migration | 2 | 17.383 | 1.646 | 0.973 |
|  | GO:0050860 | negative regulation of T cell receptor signaling pathway | 2 | 17.383 | 1.646 | 0.973 |
|  | GO:0090201 | negative regulation of release of cytochrome c from mitochondria | 2 | 17.383 | 1.646 | 0.973 |
|  | GO:0007626 | locomotory behavior | 4 | 5.624 | 1.643 | 0.970 |
|  | GO:0050727 | regulation of inflammatory response | 3 | 8.195 | 1.634 | 0.963 |
|  | GO:0050830 | defense response to Gram-positive bacterium | 3 | 8.195 | 1.634 | 0.963 |
|  | GO:0050821 | protein stabilization | 4 | 5.542 | 1.620 | 0.951 |
|  | GO:0006974 | response to DNA damage stimulus | 6 | 3.701 | 1.618 | 0.950 |
|  | GO:0001974 | blood vessel remodeling | 3 | 7.967 | 1.599 | 0.934 |
|  | GO:0006144 | purine nucleobase metabolic process | 3 | 7.967 | 1.599 | 0.934 |
|  | GO:0051726 | regulation of cell cycle | 4 | 5.463 | 1.598 | 0.934 |
|  | GO:0016567 | protein ubiquitination | 8 | 2.953 | 1.598 | 0.934 |
|  | GO:0032753 | positive regulation of interleukin-4 production | 2 | 15.935 | 1.570 | 0.910 |
|  | GO:0042832 | defense response to protozoan | 2 | 15.935 | 1.570 | 0.910 |
|  | GO:0043252 | sodium-independent organic anion transport | 2 | 15.935 | 1.570 | 0.910 |
|  | GO:0045088 | regulation of innate immune response | 2 | 15.935 | 1.570 | 0.910 |
|  | GO:0035690 | cellular response to drug | 3 | 7.752 | 1.566 | 0.908 |
|  | GO:0070936 | protein K48-linked ubiquitination | 3 | 7.752 | 1.566 | 0.908 |
|  | GO:0007265 | Ras protein signal transduction | 4 | 5.239 | 1.534 | 0.880 |
|  | GO:0030154 | cell differentiation | 10 | 2.503 | 1.521 | 0.880 |
|  | GO:0007268 | synaptic transmission | 10 | 2.490 | 1.506 | 0.880 |
|  | GO:0006865 | amino acid transport | 3 | 7.354 | 1.502 | 0.880 |
|  | GO:0031532 | actin cytoskeleton reorganization | 3 | 7.354 | 1.502 | 0.880 |
|  | GO:0001706 | endoderm formation | 2 | 14.709 | 1.501 | 0.880 |
|  | GO:0006957 | complement activation, alternative pathway | 2 | 14.709 | 1.501 | 0.880 |
|  | GO:0009311 | oligosaccharide metabolic process | 2 | 14.709 | 1.501 | 0.880 |
|  | GO:0042136 | neurotransmitter biosynthetic process | 2 | 14.709 | 1.501 | 0.880 |
|  | GO:0045445 | myoblast differentiation | 2 | 14.709 | 1.501 | 0.880 |
|  | GO:0051000 | positive regulation of nitric-oxide synthase activity | 2 | 14.709 | 1.501 | 0.880 |
|  | GO:0030308 | negative regulation of cell growth | 5 | 4.086 | 1.500 | 0.880 |
|  | GO:0007160 | cell-matrix adhesion | 4 | 5.099 | 1.494 | 0.880 |
|  | GO:0006811 | ion transport | 6 | 3.477 | 1.490 | 0.880 |
|  | GO:0030162 | regulation of proteolysis | 3 | 7.171 | 1.471 | 0.880 |
|  | GO:0045860 | positive regulation of protein kinase activity | 3 | 7.171 | 1.471 | 0.880 |
|  | GO:0050796 | regulation of insulin secretion | 4 | 4.967 | 1.454 | 0.880 |
|  | GO:0048146 | positive regulation of fibroblast proliferation | 3 | 6.996 | 1.442 | 0.880 |
|  | GO:0002407 | dendritic cell chemotaxis | 2 | 13.658 | 1.437 | 0.880 |
|  | GO:0006825 | copper ion transport | 2 | 13.658 | 1.437 | 0.880 |
|  | GO:0008299 | isoprenoid biosynthetic process | 2 | 13.658 | 1.437 | 0.880 |
|  | GO:0032781 | positive regulation of ATPase activity | 2 | 13.658 | 1.437 | 0.880 |
|  | GO:0033137 | negative regulation of peptidyl-serine phosphorylation | 2 | 13.658 | 1.437 | 0.880 |
|  | GO:0042953 | lipoprotein transport | 2 | 13.658 | 1.437 | 0.880 |
|  | GO:0045954 | positive regulation of natural killer cell mediated cytotoxicity | 2 | 13.658 | 1.437 | 0.880 |
|  | GO:0046579 | positive regulation of Ras protein signal transduction | 2 | 13.658 | 1.437 | 0.880 |
|  | GO:0007565 | female pregnancy | 4 | 4.903 | 1.435 | 0.880 |
|  | GO:0008283 | cell proliferation | 9 | 2.561 | 1.420 | 0.880 |
|  | GO:0006351 | transcription, DNA-dependent | 31 | 1.622 | 1.417 | 0.880 |
|  | GO:0006139 | nucleobase-containing compound metabolic process | 3 | 6.829 | 1.413 | 0.880 |
|  | GO:0007420 | brain development | 6 | 3.297 | 1.383 | 0.880 |
|  | GO:0000084 | S phase of mitotic cell cycle | 5 | 3.824 | 1.383 | 0.880 |
|  | GO:0000097 | sulfur amino acid biosynthetic process | 1 | 95.607 | 1.378 | 0.880 |
|  | GO:0001080 | nitrogen catabolite activation of transcription from RNA polymerase II promoter | 1 | 95.607 | 1.378 | 0.880 |
|  | GO:0001887 | selenium compound metabolic process | 1 | 95.607 | 1.378 | 0.880 |
|  | GO:0001985 | negative regulation of heart rate involved in baroreceptor response to increased systemic arterial blood pressure | 1 | 95.607 | 1.378 | 0.880 |
|  | GO:0002223 | stimulatory C-type lectin receptor signaling pathway | 1 | 95.607 | 1.378 | 0.880 |
|  | GO:0002485 | antigen processing and presentation of endogenous peptide antigen via MHC class I via ER pathway, TAP-dependent | 1 | 95.607 | 1.378 | 0.880 |
|  | GO:0002489 | antigen processing and presentation of endogenous peptide antigen via MHC class Ib via ER pathway, TAP-dependent | 1 | 95.607 | 1.378 | 0.880 |
|  | GO:0002517 | T cell tolerance induction | 1 | 95.607 | 1.378 | 0.880 |
|  | GO:0002534 | cytokine production involved in inflammatory response | 1 | 95.607 | 1.378 | 0.880 |
|  | GO:0002591 | positive regulation of antigen processing and presentation of peptide antigen via MHC class I | 1 | 95.607 | 1.378 | 0.880 |
|  | GO:0002676 | regulation of chronic inflammatory response | 1 | 95.607 | 1.378 | 0.880 |
|  | GO:0002678 | positive regulation of chronic inflammatory response | 1 | 95.607 | 1.378 | 0.880 |
|  | GO:0002685 | regulation of leukocyte migration | 1 | 95.607 | 1.378 | 0.880 |
|  | GO:0002792 | negative regulation of peptide secretion | 1 | 95.607 | 1.378 | 0.880 |
|  | GO:0006062 | sorbitol catabolic process | 1 | 95.607 | 1.378 | 0.880 |
|  | GO:0006083 | acetate metabolic process | 1 | 95.607 | 1.378 | 0.880 |
|  | GO:0006109 | regulation of carbohydrate metabolic process | 1 | 95.607 | 1.378 | 0.880 |
|  | GO:0006175 | dATP biosynthetic process | 1 | 95.607 | 1.378 | 0.880 |
|  | GO:0006535 | cysteine biosynthetic process from serine | 1 | 95.607 | 1.378 | 0.880 |
|  | GO:0009115 | xanthine catabolic process | 1 | 95.607 | 1.378 | 0.880 |
|  | GO:0009403 | toxin biosynthetic process | 1 | 95.607 | 1.378 | 0.880 |
|  | GO:0009441 | glycolate metabolic process | 1 | 95.607 | 1.378 | 0.880 |
|  | GO:0010046 | response to mycotoxin | 1 | 95.607 | 1.378 | 0.880 |
|  | GO:0010124 | phenylacetate catabolic process | 1 | 95.607 | 1.378 | 0.880 |
|  | GO:0010607 | negative regulation of cytoplasmic mRNA processing body assembly | 1 | 95.607 | 1.378 | 0.880 |
|  | GO:0010635 | regulation of mitochondrial fusion | 1 | 95.607 | 1.378 | 0.880 |
|  | GO:0010722 | regulation of ferrochelatase activity | 1 | 95.607 | 1.378 | 0.880 |
|  | GO:0014820 | tonic smooth muscle contraction | 1 | 95.607 | 1.378 | 0.880 |
|  | GO:0014896 | muscle hypertrophy | 1 | 95.607 | 1.378 | 0.880 |
|  | GO:0015787 | UDP-glucuronic acid transport | 1 | 95.607 | 1.378 | 0.880 |
|  | GO:0015789 | UDP-N-acetylgalactosamine transport | 1 | 95.607 | 1.378 | 0.880 |
|  | GO:0015874 | norepinephrine transport | 1 | 95.607 | 1.378 | 0.880 |
|  | GO:0016116 | carotenoid metabolic process | 1 | 95.607 | 1.378 | 0.880 |
|  | GO:0016119 | carotene metabolic process | 1 | 95.607 | 1.378 | 0.880 |
|  | GO:0016121 | carotene catabolic process | 1 | 95.607 | 1.378 | 0.880 |
|  | GO:0018924 | mandelate metabolic process | 1 | 95.607 | 1.378 | 0.880 |
|  | GO:0019343 | cysteine biosynthetic process via cystathionine | 1 | 95.607 | 1.378 | 0.880 |
|  | GO:0019481 | L-alanine catabolic process, by transamination | 1 | 95.607 | 1.378 | 0.880 |
|  | GO:0019482 | beta-alanine metabolic process | 1 | 95.607 | 1.378 | 0.880 |
|  | GO:0019627 | urea metabolic process | 1 | 95.607 | 1.378 | 0.880 |
|  | GO:0019859 | thymine metabolic process | 1 | 95.607 | 1.378 | 0.880 |
|  | GO:0019883 | antigen processing and presentation of endogenous antigen | 1 | 95.607 | 1.378 | 0.880 |
|  | GO:0021629 | olfactory nerve structural organization | 1 | 95.607 | 1.378 | 0.880 |
|  | GO:0022406 | membrane docking | 1 | 95.607 | 1.378 | 0.880 |
|  | GO:0030329 | prenylcysteine metabolic process | 1 | 95.607 | 1.378 | 0.880 |
|  | GO:0030887 | positive regulation of myeloid dendritic cell activation | 1 | 95.607 | 1.378 | 0.880 |
|  | GO:0031129 | inductive cell-cell signaling | 1 | 95.607 | 1.378 | 0.880 |
|  | GO:0032078 | negative regulation of endodeoxyribonuclease activity | 1 | 95.607 | 1.378 | 0.880 |
|  | GO:0032304 | negative regulation of icosanoid secretion | 1 | 95.607 | 1.378 | 0.880 |
|  | GO:0032385 | positive regulation of intracellular cholesterol transport | 1 | 95.607 | 1.378 | 0.880 |
|  | GO:0032814 | regulation of natural killer cell activation | 1 | 95.607 | 1.378 | 0.880 |
|  | GO:0032817 | regulation of natural killer cell proliferation | 1 | 95.607 | 1.378 | 0.880 |
|  | GO:0033262 | regulation of nuclear cell cycle DNA replication | 1 | 95.607 | 1.378 | 0.880 |
|  | GO:0033514 | L-lysine catabolic process to acetyl-CoA via L-pipecolate | 1 | 95.607 | 1.378 | 0.880 |
|  | GO:0033609 | oxalate metabolic process | 1 | 95.607 | 1.378 | 0.880 |
|  | GO:0034255 | regulation of urea metabolic process | 1 | 95.607 | 1.378 | 0.880 |
|  | GO:0034276 | kynurenic acid biosynthetic process | 1 | 95.607 | 1.378 | 0.880 |
|  | GO:0034633 | retinol transport | 1 | 95.607 | 1.378 | 0.880 |
|  | GO:0035042 | fertilization, exchange of chromosomal proteins | 1 | 95.607 | 1.378 | 0.880 |
|  | GO:0035428 | hexose transmembrane transport | 1 | 95.607 | 1.378 | 0.880 |
|  | GO:0035476 | angioblast cell migration | 1 | 95.607 | 1.378 | 0.880 |
|  | GO:0035509 | negative regulation of myosin-light-chain-phosphatase activity | 1 | 95.607 | 1.378 | 0.880 |
|  | GO:0035644 | phosphoanandamide dephosphorylation | 1 | 95.607 | 1.378 | 0.880 |
|  | GO:0035691 | macrophage migration inhibitory factor signaling pathway | 1 | 95.607 | 1.378 | 0.880 |
|  | GO:0035963 | cellular response to interleukin-13 | 1 | 95.607 | 1.378 | 0.880 |
|  | GO:0035998 | 7,8-dihydroneopterin 3'-triphosphate biosynthetic process | 1 | 95.607 | 1.378 | 0.880 |
|  | GO:0038114 | interleukin-21-mediated signaling pathway | 1 | 95.607 | 1.378 | 0.880 |
|  | GO:0038185 | intracellular bile acid receptor signaling pathway | 1 | 95.607 | 1.378 | 0.880 |
|  | GO:0042413 | carnitine catabolic process | 1 | 95.607 | 1.378 | 0.880 |
|  | GO:0042559 | pteridine-containing compound biosynthetic process | 1 | 95.607 | 1.378 | 0.880 |
|  | GO:0042697 | menopause | 1 | 95.607 | 1.378 | 0.880 |
|  | GO:0042790 | transcription of nuclear large rRNA transcript from RNA polymerase I promoter | 1 | 95.607 | 1.378 | 0.880 |
|  | GO:0042908 | xenobiotic transport | 1 | 95.607 | 1.378 | 0.880 |
|  | GO:0043378 | positive regulation of CD8-positive, alpha-beta T cell differentiation | 1 | 95.607 | 1.378 | 0.880 |
|  | GO:0043383 | negative T cell selection | 1 | 95.607 | 1.378 | 0.880 |
|  | GO:0043418 | homocysteine catabolic process | 1 | 95.607 | 1.378 | 0.880 |
|  | GO:0043603 | cellular amide metabolic process | 1 | 95.607 | 1.378 | 0.880 |
|  | GO:0044328 | canonical Wnt receptor signaling pathway involved in positive regulation of endothelial cell migration | 1 | 95.607 | 1.378 | 0.880 |
|  | GO:0044329 | canonical Wnt receptor signaling pathway involved in positive regulation of cell-cell adhesion | 1 | 95.607 | 1.378 | 0.880 |
|  | GO:0044330 | canonical Wnt receptor signaling pathway involved in positive regulation of wound healing | 1 | 95.607 | 1.378 | 0.880 |
|  | GO:0045065 | cytotoxic T cell differentiation | 1 | 95.607 | 1.378 | 0.880 |
|  | GO:0045204 | MAPK export from nucleus | 1 | 95.607 | 1.378 | 0.880 |
|  | GO:0045209 | MAPK phosphatase export from nucleus, leptomycin B sensitive | 1 | 95.607 | 1.378 | 0.880 |
|  | GO:0045621 | positive regulation of lymphocyte differentiation | 1 | 95.607 | 1.378 | 0.880 |
|  | GO:0045940 | positive regulation of steroid metabolic process | 1 | 95.607 | 1.378 | 0.880 |
|  | GO:0046041 | ITP metabolic process | 1 | 95.607 | 1.378 | 0.880 |
|  | GO:0046051 | UTP metabolic process | 1 | 95.607 | 1.378 | 0.880 |
|  | GO:0046164 | alcohol catabolic process | 1 | 95.607 | 1.378 | 0.880 |
|  | GO:0046177 | D-gluconate catabolic process | 1 | 95.607 | 1.378 | 0.880 |
|  | GO:0046272 | stilbene catabolic process | 1 | 95.607 | 1.378 | 0.880 |
|  | GO:0046296 | glycolate catabolic process | 1 | 95.607 | 1.378 | 0.880 |
|  | GO:0046327 | glycerol biosynthetic process from pyruvate | 1 | 95.607 | 1.378 | 0.880 |
|  | GO:0046359 | butyrate catabolic process | 1 | 95.607 | 1.378 | 0.880 |
|  | GO:0046370 | fructose biosynthetic process | 1 | 95.607 | 1.378 | 0.880 |
|  | GO:0046434 | organophosphate catabolic process | 1 | 95.607 | 1.378 | 0.880 |
|  | GO:0046479 | glycosphingolipid catabolic process | 1 | 95.607 | 1.378 | 0.880 |
|  | GO:0046491 | L-methylmalonyl-CoA metabolic process | 1 | 95.607 | 1.378 | 0.880 |
|  | GO:0046618 | drug export | 1 | 95.607 | 1.378 | 0.880 |
|  | GO:0046633 | alpha-beta T cell proliferation | 1 | 95.607 | 1.378 | 0.880 |
|  | GO:0046724 | oxalic acid secretion | 1 | 95.607 | 1.378 | 0.880 |
|  | GO:0046940 | nucleoside monophosphate phosphorylation | 1 | 95.607 | 1.378 | 0.880 |
|  | GO:0048241 | epinephrine transport | 1 | 95.607 | 1.378 | 0.880 |
|  | GO:0048313 | Golgi inheritance | 1 | 95.607 | 1.378 | 0.880 |
|  | GO:0050666 | regulation of homocysteine metabolic process | 1 | 95.607 | 1.378 | 0.880 |
|  | GO:0050857 | positive regulation of antigen receptor-mediated signaling pathway | 1 | 95.607 | 1.378 | 0.880 |
|  | GO:0050859 | negative regulation of B cell receptor signaling pathway | 1 | 95.607 | 1.378 | 0.880 |
|  | GO:0051066 | dihydrobiopterin metabolic process | 1 | 95.607 | 1.378 | 0.880 |
|  | GO:0051126 | negative regulation of actin nucleation | 1 | 95.607 | 1.378 | 0.880 |
|  | GO:0051160 | L-xylitol catabolic process | 1 | 95.607 | 1.378 | 0.880 |
|  | GO:0051164 | L-xylitol metabolic process | 1 | 95.607 | 1.378 | 0.880 |
|  | GO:0051249 | regulation of lymphocyte activation | 1 | 95.607 | 1.378 | 0.880 |
|  | GO:0051459 | regulation of corticotropin secretion | 1 | 95.607 | 1.378 | 0.880 |
|  | GO:0051460 | negative regulation of corticotropin secretion | 1 | 95.607 | 1.378 | 0.880 |
|  | GO:0051531 | NFAT protein import into nucleus | 1 | 95.607 | 1.378 | 0.880 |
|  | GO:0051615 | histamine uptake | 1 | 95.607 | 1.378 | 0.880 |
|  | GO:0051793 | medium-chain fatty acid catabolic process | 1 | 95.607 | 1.378 | 0.880 |
|  | GO:0060550 | positive regulation of fructose 1,6-bisphosphate 1-phosphatase activity | 1 | 95.607 | 1.378 | 0.880 |
|  | GO:0060552 | positive regulation of fructose 1,6-bisphosphate metabolic process | 1 | 95.607 | 1.378 | 0.880 |
|  | GO:0060554 | activation of necroptosis of activated-T cells | 1 | 95.607 | 1.378 | 0.880 |
|  | GO:0060809 | mesodermal to mesenchymal transition involved in gastrulation | 1 | 95.607 | 1.378 | 0.880 |
|  | GO:0060857 | establishment of glial blood-brain barrier | 1 | 95.607 | 1.378 | 0.880 |
|  | GO:0060988 | lipid tube assembly | 1 | 95.607 | 1.378 | 0.880 |
|  | GO:0061025 | membrane fusion | 1 | 95.607 | 1.378 | 0.880 |
|  | GO:0061326 | renal tubule development | 1 | 95.607 | 1.378 | 0.880 |
|  | GO:0070094 | positive regulation of glucagon secretion | 1 | 95.607 | 1.378 | 0.880 |
|  | GO:0070228 | regulation of lymphocyte apoptotic process | 1 | 95.607 | 1.378 | 0.880 |
|  | GO:0070346 | positive regulation of fat cell proliferation | 1 | 95.607 | 1.378 | 0.880 |
|  | GO:0070489 | T cell aggregation | 1 | 95.607 | 1.378 | 0.880 |
|  | GO:0070541 | response to platinum ion | 1 | 95.607 | 1.378 | 0.880 |
|  | GO:0071351 | cellular response to interleukin-18 | 1 | 95.607 | 1.378 | 0.880 |
|  | GO:0071417 | cellular response to organic nitrogen | 1 | 95.607 | 1.378 | 0.880 |
|  | GO:0071615 | oxidative deethylation | 1 | 95.607 | 1.378 | 0.880 |
|  | GO:0071931 | positive regulation of transcription involved in G1/S phase of mitotic cell cycle | 1 | 95.607 | 1.378 | 0.880 |
|  | GO:0072073 | kidney epithelium development | 1 | 95.607 | 1.378 | 0.880 |
|  | GO:0072229 | metanephric proximal convoluted tubule development | 1 | 95.607 | 1.378 | 0.880 |
|  | GO:0072678 | T cell migration | 1 | 95.607 | 1.378 | 0.880 |
|  | GO:0090085 | regulation of protein deubiquitination | 1 | 95.607 | 1.378 | 0.880 |
|  | GO:0090107 | regulation of high-density lipoprotein particle assembly | 1 | 95.607 | 1.378 | 0.880 |
|  | GO:0090287 | regulation of cellular response to growth factor stimulus | 1 | 95.607 | 1.378 | 0.880 |
|  | GO:0090298 | negative regulation of mitochondrial DNA replication | 1 | 95.607 | 1.378 | 0.880 |
|  | GO:0090361 | regulation of platelet-derived growth factor production | 1 | 95.607 | 1.378 | 0.880 |
|  | GO:1900011 | negative regulation of corticotropin-releasing hormone receptor activity | 1 | 95.607 | 1.378 | 0.880 |
|  | GO:1901373 | lipid hydroperoxide transport | 1 | 95.607 | 1.378 | 0.880 |
|  | GO:2000169 | regulation of peptidyl-cysteine S-nitrosylation | 1 | 95.607 | 1.378 | 0.880 |
|  | GO:2000213 | positive regulation of glutamate metabolic process | 1 | 95.607 | 1.378 | 0.880 |
|  | GO:2000564 | regulation of CD8-positive, alpha-beta T cell proliferation | 1 | 95.607 | 1.378 | 0.880 |
|  | GO:2000672 | negative regulation of motor neuron apoptotic process | 1 | 95.607 | 1.378 | 0.880 |
|  | GO:2001213 | negative regulation of vasculogenesis | 1 | 95.607 | 1.378 | 0.880 |
|  | GO:2001250 | positive regulation of ammonia assimilation cycle | 1 | 95.607 | 1.378 | 0.880 |
|  | GO:2001294 | malonyl-CoA catabolic process | 1 | 95.607 | 1.378 | 0.880 |
|  | GO:0002230 | positive regulation of defense response to virus by host | 2 | 12.748 | 1.378 | 0.880 |
|  | GO:0030048 | actin filament-based movement | 2 | 12.748 | 1.378 | 0.880 |
|  | GO:0030449 | regulation of complement activation | 2 | 12.748 | 1.378 | 0.880 |
|  | GO:0035265 | organ growth | 2 | 12.748 | 1.378 | 0.880 |
|  | GO:0042246 | tissue regeneration | 2 | 12.748 | 1.378 | 0.880 |
|  | GO:0050930 | induction of positive chemotaxis | 2 | 12.748 | 1.378 | 0.880 |
|  | GO:0060749 | mammary gland alveolus development | 2 | 12.748 | 1.378 | 0.880 |
|  | GO:0016032 | viral reproduction | 9 | 2.516 | 1.373 | 0.876 |
|  | GO:0038096 | Fc-gamma receptor signaling pathway involved in phagocytosis | 4 | 4.664 | 1.360 | 0.864 |
|  | GO:0006520 | cellular amino acid metabolic process | 3 | 6.519 | 1.358 | 0.863 |
|  | GO:0006687 | glycosphingolipid metabolic process | 3 | 6.519 | 1.358 | 0.863 |
|  | GO:0006096 | glycolysis | 3 | 6.374 | 1.331 | 0.837 |
|  | GO:0001659 | temperature homeostasis | 2 | 11.951 | 1.323 | 0.835 |
|  | GO:0001782 | B cell homeostasis | 2 | 11.951 | 1.323 | 0.835 |
|  | GO:0006103 | 2-oxoglutarate metabolic process | 2 | 11.951 | 1.323 | 0.835 |
|  | GO:0032733 | positive regulation of interleukin-10 production | 2 | 11.951 | 1.323 | 0.835 |
|  | GO:0048514 | blood vessel morphogenesis | 2 | 11.951 | 1.323 | 0.835 |
|  | GO:0050870 | positive regulation of T cell activation | 2 | 11.951 | 1.323 | 0.835 |
|  | GO:0060334 | regulation of interferon-gamma-mediated signaling pathway | 2 | 11.951 | 1.323 | 0.835 |
|  | GO:0071158 | positive regulation of cell cycle arrest | 2 | 11.951 | 1.323 | 0.835 |
|  | GO:0090023 | positive regulation of neutrophil chemotaxis | 2 | 11.951 | 1.323 | 0.835 |
|  | GO:0014068 | positive regulation of phosphatidylinositol 3-kinase cascade | 3 | 6.235 | 1.305 | 0.818 |
| **Pathway** | 01100 | Metabolic pathways | 116 | 8.987 | 72.555 | 70.142 |
|  | 00280 | "Valine, leucine and isoleucine degradation" | 21 | 42.718 | 28.145 | 26.033 |
|  | 00071 | Fatty acid degradation | 18 | 39.112 | 23.249 | 21.313 |
|  | 00640 | Propanoate metabolism | 14 | 41.828 | 18.589 | 16.778 |
|  | 04612 | Antigen processing and presentation | 18 | 22.350 | 18.187 | 16.473 |
|  | 04146 | Peroxisome | 18 | 20.734 | 17.552 | 15.917 |
|  | 01200 | Carbon metabolism | 19 | 16.076 | 16.314 | 14.746 |
|  | 03320 | PPAR signaling pathway | 16 | 22.170 | 16.109 | 14.650 |
|  | 04610 | Complement and coagulation cascades | 16 | 22.170 | 16.109 | 14.650 |
|  | 00650 | Butanoate metabolism | 12 | 42.492 | 16.031 | 14.618 |
|  | 05164 | Influenza A | 20 | 10.927 | 13.807 | 12.435 |
|  | 00830 | Retinol metabolism | 14 | 20.592 | 13.615 | 12.281 |
|  | 05332 | Graft-versus-host disease | 12 | 27.983 | 13.432 | 12.132 |
|  | 00410 | beta-Alanine metabolism | 11 | 33.925 | 13.376 | 12.109 |
|  | 05150 | Staphylococcus aureus infection | 13 | 22.598 | 13.211 | 11.974 |
|  | 04660 | T cell receptor signaling pathway | 16 | 14.709 | 13.113 | 11.904 |
|  | 01212 | Fatty acid metabolism | 12 | 23.902 | 12.515 | 11.343 |
|  | 05322 | Systemic lupus erythematosus | 17 | 12.129 | 12.501 | 11.343 |
|  | 05330 | Allograft rejection | 11 | 28.424 | 12.396 | 11.262 |
|  | 05168 | Herpes simplex infection | 19 | 9.872 | 12.311 | 11.198 |
|  | 05321 | Inflammatory bowel disease (IBD) | 13 | 19.121 | 12.198 | 11.107 |
|  | 00260 | "Glycine, serine and threonine metabolism" | 11 | 26.292 | 11.976 | 10.925 |
|  | 00380 | Tryptophan metabolism | 11 | 26.292 | 11.976 | 10.925 |
|  | 00982 | Drug metabolism - cytochrome P450 | 13 | 18.278 | 11.930 | 10.897 |
|  | 04940 | Type I diabetes mellitus | 11 | 24.458 | 11.593 | 10.578 |
|  | 05145 | Toxoplasmosis | 15 | 12.153 | 11.049 | 10.051 |
|  | 05340 | Primary immunodeficiency | 10 | 26.558 | 10.935 | 9.953 |
|  | 05204 | Chemical carcinogenesis | 13 | 15.344 | 10.906 | 9.940 |
|  | 05320 | Autoimmune thyroid disease | 11 | 20.225 | 10.609 | 9.658 |
|  | 00630 | Glyoxylate and dicarboxylate metabolism | 9 | 30.731 | 10.485 | 9.549 |
|  | 04650 | Natural killer cell mediated cytotoxicity | 15 | 10.702 | 10.238 | 9.316 |
|  | 05416 | Viral myocarditis | 11 | 18.132 | 10.057 | 9.149 |
|  | 04514 | Cell adhesion molecules (CAMs) | 15 | 10.099 | 9.873 | 8.978 |
|  | 00010 | Glycolysis / Gluconeogenesis | 10 | 14.270 | 8.072 | 7.190 |
|  | 05140 | Leishmaniasis | 10 | 13.279 | 7.757 | 6.888 |
|  | 00620 | Pyruvate metabolism | 8 | 19.121 | 7.503 | 6.646 |
|  | 04152 | AMPK signaling pathway | 12 | 9.252 | 7.469 | 6.624 |
|  | 04145 | Phagosome | 13 | 8.123 | 7.399 | 6.565 |
|  | 04062 | Chemokine signaling pathway | 14 | 7.158 | 7.258 | 6.436 |
|  | 00120 | Primary bile acid biosynthesis | 6 | 33.744 | 7.241 | 6.430 |
|  | 04068 | FoxO signaling pathway | 12 | 8.562 | 7.088 | 6.287 |
|  | 05310 | Asthma | 7 | 22.308 | 7.058 | 6.268 |
|  | 04931 | Insulin resistance | 11 | 9.648 | 7.039 | 6.259 |
|  | 00983 | Drug metabolism - other enzymes | 8 | 16.627 | 6.997 | 6.227 |
|  | 04672 | Intestinal immune network for IgA production | 8 | 16.274 | 6.919 | 6.159 |
|  | 05323 | Rheumatoid arthritis | 10 | 10.742 | 6.849 | 6.099 |
|  | 05152 | Tuberculosis | 13 | 7.022 | 6.644 | 5.902 |
|  | 00232 | Caffeine metabolism | 4 | 76.486 | 6.629 | 5.897 |
|  | 00310 | Lysine degradation | 8 | 14.709 | 6.560 | 5.844 |
|  | 00350 | Tyrosine metabolism | 7 | 19.121 | 6.559 | 5.844 |
|  | 05166 | HTLV-I infection | 15 | 5.559 | 6.312 | 5.607 |
|  | 05142 | Chagas disease (American trypanosomiasis) | 10 | 9.193 | 6.200 | 5.503 |
|  | 04910 | Insulin signaling pathway | 11 | 7.566 | 5.950 | 5.261 |
|  | 04917 | Prolactin signaling pathway | 8 | 10.623 | 5.439 | 4.758 |
|  | 04060 | Cytokine-cytokine receptor interaction | 14 | 5.051 | 5.396 | 4.723 |
|  | 00980 | Metabolism of xenobiotics by cytochrome P450 | 8 | 10.336 | 5.346 | 4.681 |
|  | 05162 | Measles | 10 | 7.135 | 5.176 | 4.519 |
|  | 00072 | Synthesis and degradation of ketone bodies | 4 | 38.243 | 5.024 | 4.374 |
|  | 00140 | Steroid hormone biosynthesis | 7 | 11.539 | 4.998 | 4.356 |
|  | 00340 | Histidine metabolism | 5 | 20.784 | 4.848 | 4.213 |
|  | 00590 | Arachidonic acid metabolism | 7 | 10.794 | 4.800 | 4.172 |
|  | 00860 | Porphyrin and chlorophyll metabolism | 6 | 13.658 | 4.710 | 4.090 |
|  | 04666 | Fc gamma R-mediated phagocytosis | 8 | 8.314 | 4.627 | 4.013 |
|  | 02010 | ABC transporters | 6 | 13.037 | 4.589 | 3.982 |
|  | 04976 | Bile secretion | 7 | 9.426 | 4.404 | 3.803 |
|  | 04922 | Glucagon signaling pathway | 8 | 7.573 | 4.326 | 3.736 |
|  | 00030 | Pentose phosphate pathway | 5 | 16.484 | 4.323 | 3.736 |
|  | 05133 | Pertussis | 7 | 8.923 | 4.245 | 3.665 |
|  | 04620 | Toll-like receptor signaling pathway | 8 | 7.216 | 4.172 | 3.598 |
|  | 04726 | Serotonergic synapse | 8 | 6.829 | 3.998 | 3.430 |
|  | 00500 | Starch and sucrose metabolism | 6 | 10.244 | 3.973 | 3.411 |
|  | 05020 | Prion diseases | 5 | 13.658 | 3.909 | 3.353 |
|  | 04630 | Jak-STAT signaling pathway | 9 | 5.446 | 3.719 | 3.169 |
|  | 00900 | Terpenoid backbone biosynthesis | 4 | 17.383 | 3.525 | 2.981 |
|  | 00270 | Cysteine and methionine metabolism | 5 | 10.864 | 3.418 | 2.880 |
|  | 05231 | Choline metabolism in cancer | 7 | 6.626 | 3.409 | 2.877 |
|  | 01230 | Biosynthesis of amino acids | 6 | 7.752 | 3.285 | 2.758 |
|  | 00053 | Ascorbate and aldarate metabolism | 4 | 14.164 | 3.163 | 2.647 |
|  | 04320 | Dorso-ventral axis formation | 4 | 14.164 | 3.163 | 2.647 |
|  | 00330 | Arginine and proline metabolism | 5 | 9.561 | 3.150 | 2.640 |
|  | 00591 | Linoleic acid metabolism | 4 | 13.187 | 3.039 | 2.534 |
|  | 04919 | Thyroid hormone signaling pathway | 7 | 5.672 | 2.989 | 2.489 |
|  | 00020 | Citrate cycle (TCA cycle) | 4 | 12.748 | 2.980 | 2.486 |
|  | 04640 | Hematopoietic cell lineage | 6 | 6.594 | 2.899 | 2.410 |
|  | 04010 | MAPK signaling pathway | 10 | 3.749 | 2.797 | 2.313 |
|  | 05143 | African trypanosomiasis | 4 | 11.248 | 2.766 | 2.287 |
|  | 04810 | Regulation of actin cytoskeleton | 9 | 4.021 | 2.730 | 2.256 |
|  | 00250 | "Alanine, aspartate and glutamate metabolism" | 4 | 10.927 | 2.717 | 2.250 |
|  | 04380 | Osteoclast differentiation | 7 | 5.109 | 2.714 | 2.250 |
|  | 00040 | Pentose and glucuronate interconversions | 4 | 10.623 | 2.669 | 2.210 |
|  | 05212 | Pancreatic cancer | 5 | 7.243 | 2.584 | 2.130 |
|  | 00360 | Phenylalanine metabolism | 3 | 16.872 | 2.556 | 2.107 |
|  | 00770 | Pantothenate and CoA biosynthesis | 3 | 15.935 | 2.481 | 2.036 |
|  | 04920 | Adipocytokine signaling pathway | 5 | 6.829 | 2.468 | 2.027 |
|  | 04975 | Fat digestion and absorption | 4 | 9.328 | 2.452 | 2.016 |
|  | 04151 | PI3K-Akt signaling pathway | 11 | 3.048 | 2.335 | 1.904 |
|  | 05169 | Epstein-Barr virus infection | 8 | 3.824 | 2.281 | 1.854 |
|  | 05203 | Viral carcinogenesis | 8 | 3.731 | 2.213 | 1.791 |
|  | 04350 | TGF-beta signaling pathway | 5 | 5.691 | 2.113 | 1.695 |
|  | 04110 | Cell cycle | 6 | 4.626 | 2.093 | 1.680 |
|  | 00062 | Fatty acid elongation | 3 | 11.473 | 2.054 | 1.645 |
|  | 04727 | GABAergic synapse | 5 | 5.432 | 2.024 | 1.619 |
|  | 05215 | Prostate cancer | 5 | 5.371 | 2.003 | 1.602 |
|  | 04080 | Neuroactive ligand-receptor interaction | 9 | 3.106 | 1.953 | 1.557 |
|  | 05160 | Hepatitis C | 6 | 4.313 | 1.941 | 1.551 |
|  | 04014 | Ras signaling pathway | 8 | 3.369 | 1.939 | 1.551 |
|  | 00561 | Glycerolipid metabolism | 4 | 6.482 | 1.863 | 1.480 |
|  | 04550 | Signaling pathways regulating pluripotency of stem cells | 6 | 4.040 | 1.801 | 1.422 |
|  | 00051 | Fructose and mannose metabolism | 3 | 8.963 | 1.744 | 1.371 |
|  | 05161 | Hepatitis B | 6 | 3.929 | 1.743 | 1.371 |
|  | 04623 | Cytosolic DNA-sensing pathway | 4 | 5.975 | 1.736 | 1.368 |
|  | 05214 | Glioma | 4 | 5.884 | 1.712 | 1.348 |
|  | 04664 | Fc epsilon RI signaling pathway | 4 | 5.708 | 1.665 | 1.305 |
|  | 00430 | Taurine and hypotaurine metabolism | 2 | 17.383 | 1.646 | 1.290 |
|  | 04115 | p53 signaling pathway | 4 | 5.624 | 1.643 | 1.290 |
|  | 04668 | TNF signaling pathway | 5 | 4.346 | 1.610 | 1.263 |
|  | 04144 | Endocytosis | 8 | 2.965 | 1.608 | 1.263 |
|  | 03030 | DNA replication | 3 | 7.967 | 1.599 | 1.258 |
|  | 04510 | Focal adhesion | 7 | 3.233 | 1.589 | 1.251 |
|  | 04921 | Oxytocin signaling pathway | 6 | 3.631 | 1.579 | 1.246 |
|  | 05218 | Melanoma | 4 | 5.386 | 1.577 | 1.246 |
|  | 04662 | B cell receptor signaling pathway | 4 | 5.312 | 1.555 | 1.228 |
|  | 04015 | Rap1 signaling pathway | 7 | 3.172 | 1.545 | 1.222 |
|  | 04140 | Regulation of autophagy | 3 | 7.354 | 1.502 | 1.184 |
|  | 00061 | Fatty acid biosynthesis | 2 | 14.709 | 1.501 | 1.184 |
|  | 04071 | Sphingolipid signaling pathway | 5 | 3.984 | 1.455 | 1.142 |
|  | 00790 | Folate biosynthesis | 2 | 13.658 | 1.437 | 1.127 |
|  | 05200 | Pathways in cancer | 10 | 2.408 | 1.411 | 1.105 |
|  | 03050 | Proteasome | 3 | 6.519 | 1.358 | 1.055 |
|  | 05034 | Alcoholism | 6 | 3.205 | 1.327 | 1.031 |
|  | 05202 | Transcriptional misregulation in cancer | 6 | 3.205 | 1.327 | 1.031 |
